# Cell type-specific over-expression of chromosome 21 genes in fibroblasts and fetal hearts with trisomy 21

**DOI:** 10.1186/1471-2350-7-24

**Published:** 2006-03-15

**Authors:** Chi-Ming Li, Meirong Guo, Martha Salas, Nicole Schupf, Wayne Silverman, Warren B Zigman, Sameera Husain, Dorothy Warburton, Harshwardhan Thaker, Benjamin Tycko

**Affiliations:** 1 Institute for Cancer Genetics, Columbia University College of Physicians and Surgeons, New York, NY, USA; 2 Taub Institute for Research on Alzheimer's Disease and the Aging Brain, Columbia University College of Physicians and Surgeons, New York, NY, USA; 3 Gertrude H. Sergievsky Center, Columbia University College of Physicians and Surgeons, New York, NY, USA; 4 Department of Psychology, New York State Institute for Basic Research in Developmental Disabilities, New York, NY, USA; 5 Department of Pathology, Columbia University College of Physicians and Surgeons, New York, NY, USA; 6 Department of Genetics and Development, Columbia University College of Physicians and Surgeons, New York, NY, USA

## Abstract

**Background:**

Down syndrome (DS) is caused by trisomy 21 (+21), but the aberrations in gene expression resulting from this chromosomal aneuploidy are not yet completely understood.

**Methods:**

We used oligonucleotide microarrays to survey mRNA expression in early- and late-passage control and +21 fibroblasts and mid-gestation fetal hearts. We supplemented this analysis with northern blotting, western blotting, real-time RT-PCR, and immunohistochemistry.

**Results:**

We found chromosome 21 genes consistently over-represented among the genes over-expressed in the +21 samples. However, these sets of over-expressed genes differed across the three cell/tissue types. The chromosome 21 gene *MX1 *was strongly over-expressed (mean 16-fold) in senescent +21 fibroblasts, a result verified by northern and western blotting. *MX1 *is an interferon target gene, and its mRNA was induced by interferons present in +21 fibroblast conditioned medium, suggesting an autocrine loop for its over-expression. By immunohistochemistry the p78^MX1 ^protein was induced in lesional tissue of alopecia areata, an autoimmune disorder associated with DS. We found strong over-expression of the purine biosynthesis gene *GART *(mean 3-fold) in fetal hearts with +21 and verified this result by northern blotting and real-time RT-PCR.

**Conclusion:**

Different subsets of chromosome 21 genes are over-expressed in different cell types with +21, and for some genes this over-expression is non-linear (>1.5X). Hyperactive interferon signaling is a candidate pathway for cell senescence and autoimmune disorders in DS, and abnormal purine metabolism should be investigated for a potential role in cardiac defects.

## Background

Trisomy 21 (+21) is the most common human chromosomal aneuploidy at term, and the only one with long-term viability. This syndrome has attracted intense research interest as a prevalent cause of mental retardation, and adults with DS develop pathological and neuropsychiatric aspects of Alzheimer's disease as early as age 40. DS is also associated with phenotypes outside of the central nervous system. Among these are cardiac septal defects, autoimmune diseases, and signs of premature cellular senescence including skin wrinkling. A unifying explanation for the diverse features of DS is not apparent, although they are presumably due to abnormal expression of specific sets of critical genes, both on chromosome 21 and, through gene-gene interactions, elsewhere in the genome.

Attempts to define a DS "critical region" on chromosome 21 by correlating clinical phenotypes with the locations of sub-chromosomal segments duplicated in individuals with chromosome 21 unbalanced translocations have been somewhat successful, but the critical region for the complete syndrome still encompasses most of the q-arm of this chromosome [1-3]. Simplistically, a 1.5-fold increase in expression of multiple chromosome 21 genes might be expected in DS, and gene expression in DS tissues generally but not invariably correlates with gene dosage, with the most straightforward and best documented correlations found not in primary human tissues, but in a well-controlled segmental trisomy 16 mouse model of DS [4-12], reviewed in [13]. An alternative hypothesis is that there might be non-linear changes in expression of genes on chromosome 21, and on other chromosomes, because of gene-gene interactions in the pathological genetic background of +21. According to this model, genes non-linearly altered in their expression would be strong candidates for contributing to the DS phenotype. Here we show data relevant to these issues from a screen for altered gene expression in early and late-passage fibroblasts and fetal hearts with +21.

## Methods

### Cells, tissues and RNA

Five normal and six DS skin fibroblasts were obtained from the American Type Culture Collection (Rockville, MD), from Coriell Laboratories (Camden, NJ), or as de-identified samples from the cytogenetics laboratory, stored by the Columbia University Pathology Department Tissue Bank. All of the cell were maintained and passaged at a ratio of 1:4 in DMEM (Invitrogen, Carlsbad, CA) containing 10% FBS (Sigma-Aldrich, St. Louis, MO). Cell senescence was scored by activity of acid β-galactosidase every 3 to 5 generations. Tissue from fetal hearts at 15 to 23 weeks gestation, from cases of +21 and controls, was obtained as de-identified cryopreserved samples from the Columbia University Pathology Department Tissue Bank. Total RNA was prepared by a two-step procedure using Trizol™ reagent (Invitrogen), followed by RNeasy (Qiagen, Valencia, CA) purification. This work was carried out in compliance with the Helsinki Declaration: since the human fibroblast and heart tissues were de-identified samples, this study was not considered human subjects research by the Columbia University Internal Review Board.

### Northern blotting

Total RNA was resolved on formaldehyde-containing agarose gels and transferred to Nytran membranes (Schleicher and Schull, Keene, NH). The probes for northern blotting were partial cDNAs prepared from RT-PCR using gene-specific primers. Hybridization was in ULTRAhyb buffer (Ambion) at 42°C degrees. Probes were stripped by boiling the membranes in 0.1 % SDS/0.1 × SSC for 2 minutes, or were allowed to decay without stripping.

### Real-time RT-PCR

Total RNA, 2 μg, was reverse transcribed with oligo-dT primers using Superscript version II reverse transcriptase (Invitrogen). Quantitative real-time RT-PCR was performed with the Master SYBR Green I system (Roche Molecular Biochemicals, Mannheim, Germany) in a LightCycler apparatus (Roche) with an initial denaturation at 95°C for 30 seconds followed by multiple cycles of denaturation at 95°C for 5 seconds, primer annealing at 62°C for 5 seconds, and extension at 72°C for 12 seconds. LightCycler Software 4.0 (Roche) was used for data analysis, with mRNA content determined from inflection points of SYBR Green fluorescence plotted as a function of cycle number. The following oligonucleotide primers were used for the PCR reactions: *GART*, sense primer 5'-CCATAGCTTTCCTCCAGCAG-3' and anti-sense primer 5'-AGTTCCAACGCCATCTGTTC-3'; *HPRT *(housekeeping gene control), sense primer 5'-TTGCTCGAGATGTGATGAAGGA-3' and anti-sense primer 5'-TTCCAGTTAAAGTGGAGAGATCA-3'.

### Western blotting

After boiling for 10 minutes in a denaturing solution containing 12 mM Tri pH6.8, 5% glycerol, 0.4% SDS, 3 mM 2-mercaptomethanol, and 0.02% bromophenol blue, 30 μg of total protein lysates from +21 and control fibroblasts were subjected to electrophoresis on SDS-denaturating 4–20 % polyacylamide gels (Invitrogen). After transferring to the nitrocellulose membrane and blocking by 5% milk, the membrane was hybridized with mouse antibodies against p78^MX1 ^or α-actin (Santa Cruz Biotechnology, Santa Cruz, CA) in a solution containing 0.1% BSA, 0.1% Tween-20, and 3% dry milk overnight at 4°C. After washing, the signal was amplified using a peroxidase-conjugated goat anti-mouse IgG (Amersham Pharmarcia Biotech, Piscataway, NJ) and was visualized using a commercial ECL plus detection system (Amersham Pharmarcia Biotech).

### Microarray screening and data analysis

HGU95A GeneChips were purchased from Affymetrix (Santa Clara, CA). Double stranded cDNA was generated from 5 μg of total RNA using a poly-dT oligonucleotide that contained a T7 RNA polymerase initiation site and the Superscript Choice System Kit (Invitrogen). The cDNA was phenol/chloroform extracted, and biotinylated cRNA was generated by in vitro transcription using the Bio Array High Yield RNA Transcript Labeling System (Enzo, Farmingdale, NY). The cRNA was purified using RNeasy. cRNA was fragmented according to the Affymetrix protocol, and 15 μg of biotinylated cRNA were hybridized to U95A microarrays (Affymetrix). After scanning, the expression values for each gene were determined using Affymetrix GeneChip software v. 5.0. For each sample, the signals for each gene (probe set) were normalized to the values for the entire microarray, using GeneSpring software (Silicon Genetics, Redwood City, CA). Statistical analyses and automated clustering of the microarray data were done using the Filter Genes and Gene Tree functions of GeneSpring. For supervised analysis, we used ANOVA to identify all genes that differed at p < .05 and, in a separate analysis, at p < .01, in a given cell type (early-passage fibroblast, late-passage fibroblast, fetal hearts) as a function of the presence or absence of +21. The resulting small sets of genes were then further filtered for data quality by requiring Affymetrix 'presence calls' (high-reliability hybridization data) in at least 3 samples, and were filtered a third time for magnitude of the differences in hybridization signals by requiring at least 3 samples with signals >1.2-fold above the experiment mean. We did not correct statistically for multiple comparisons (i.e. 12,000 probe sets queried in each experiment) since doing so would reduce the statistical power far below the level needed to detect changes in the 1.5-fold range. We used the Gene Tree function to group the differentially expressed genes according to the extent of similarity in their patterns of expression across the samples, thereby separating the genes into over-expressed and under-expressed clades. Fold over-expression of each gene was calculated as the mean of the normalized signals for the +21 samples divided by the mean of the control samples.

### Antibodies and immunohistochemistry

Sections of archival formaldhehyde-fixed scalp biopsies were deparaffinized in xylene and hydrated through graded ethanols. Antigen retrieval was carried out in 1 mM EDTA buffer by boiling the slides in a microwave oven for 8 minutes at the maximum power and, sequentially, boiling for 15 minutes at a reduced power. Endogenous biotin was blocked by two steps of incubation for 10–20 minutes with egg white and 5% skim milk in 1× Tris-buffered saline (TBS) containing 0.5% BSA and 0.1 % NaN3, respectively. Between the two blocking step for biotin, slides were treated with 0.3% hydrogen peroxide in 0.1% NaN3 to block endogenous peroxidase activity. Slides were washed three times in 1× TBS buffer containing 0.1% Tween-20 (TBS-T) and then incubated with 5% of the blocking serum for 30 minutes in a humidified chamber. After 3 TBS-T washes, slides were hybridized with a monoclonal antibody against p78^MX1 ^at room temperature overnight. After washing in TBS-T, slides were incubated with biotinylated secondary anti-mouse IgG antibody (Vector Laboratories, Burlingame, CA) for 30–40 minutes at room temperature. Antigen-antibody complexes were developed using the Vectastain ABC kit (Vector Laboratories) and a chromogenic substrate, diaminobezidine (Dako). Sections were lightly counterstained with hematoxylin. For assessing the effects of interferon in +21 conditioned medium, we used a goat anti-beta-interferon antiserum (Sigma-Aldrich).

## Results

### Expression profiling in early- and late-passage fibroblasts with and without +21

**Figure 1:**
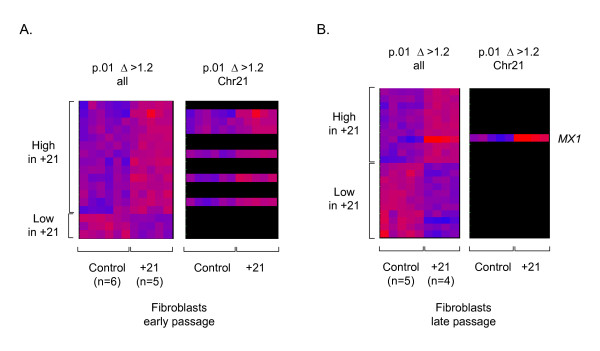
**Differential gene expression in control vs. +21 fibroblasts**. Results from supervised hierarchical clustering of the U95A microarray data are shown; with statistical criteria for selecting the genes indicated at the top (ANOVA, see Methods). Genes (probe sets) are on the x-axis and samples are on the y-axis, with expression indicated by the color scale from 0 (blue) to 5 (red), relative to the experiment mean. **A, **Data from early passage cells. As shown in the panel on the right, in which genes not on chromosome 21 have been blacked out, the set of over-expressed genes is enriched in genes on chromosome 21 and no genes on chromosome 21 are in the under-expressed set. **B, **Data from late passage cells. The *MX1 *gene is markedly over-expressed as the +21 fibroblasts become senescent. See Tables 2 and 3 for the complete lists of differentially expressed genes.

For mRNA expression profiling we used Affymetrix U95A GeneChips, which query ~10,000 human genes (12,000 oligonucleotide probe sets), including 108 unique genes (143 probe sets) from chromosome 21. Data obtained using these microarrays in our laboratory have previously shown highly reliable correlations with independent measures of gene expression, including northern blotting [14,15]. We analyzed mRNA from an accessible cell type, early- and late-passage fibroblasts, with and without +21. The growth characteristics of the fibroblast cultures are listed in Table 1. This table shows the percentages of cells staining for acid beta-galactosidase, a marker that scores positively in senescent cells and negatively in pre-senescent cells. We first asked whether there might be strong and widespread changes in gene expression due to the extra copy of chromosome 21 in the early and/or in late passage fibroblasts. Inspection of the results of non-supervised hierarchical clustering of the expression data argued against this, since this procedure did not detect the presence or absence of +21 (data not shown). We therefore did a supervised analysis of the data, attempting to identify small subsets of genes that might differ significantly in their expression as a function of the presence or absence of +21. As shown in Figure 1 and Tables 2 and 3, ANOVA with a cutoff of p < .01 yielded small sets of genes that were 'high in +21' or 'low in +21'. We repeated the ANOVA using a less stringent cutoff of p < .05, and the resulting larger gene lists are shown as supplemental tables [see Additional file 1 and Additional file 2].

**Table 1:** Characteristics of cultured fibroblasts.

**Cell Line**	**Early Passage**			**Late Passage**		
	
	**Passage no.**	**Doubling time (d)**	**beta-gal % pos.**	**Passage no.**	**Doubling time (d)**	**beta-gal % pos.**
DS1	13	2	<1	23	5.5	63.4
DS2	7	1	<1	12	19.5	37.2
DS3	4	2.5	<1	11	9	41.0
DS4	10	1.5	<1	14	8	47.5
DS5	3	5	nd	nd	nd	nd
C1	12	2	<1	24	12	55.5
C2	9	1.5	6.7	15	21	63.3
C3	5	1.5	<1	11	9.5	46.2
C4	10	2.5	<1	14	4.5	34.0
C5	3	2	n.d.	n.d.	n.d.	n.d.
C6	3	5	n.d.	n.d.	n.d.	n.d.

**Table 2:** Genes over-expressed in early passage +21 fibroblasts passing ANOVA at p < .01. Genes on chromosome 21 are in bold.

**Affy ID**	**Gene name**	**Genbank**	**Chromosome**	**Description**	**Fold change**
**36040_at**	***SH3BGR***	AI337192	**21q22.3**	**SH3 domain binding glutamic acid-rich protein**	**2.52**
39366_at	*PPP1R3C*	N36638	10q23-q24	Protein phosphatase 1, regulatory subunit 3C	1.99
31840_at	*IDE*	M21188	10q23-q25	Insulin-degrading enzyme	1.98
34542_at	*GAPDH2*	AJ005371	19q13	glyceraldehyde-3-phosphate dehydrogenase 2	1.93
**37555_at**	***PWP2***	X95263	**21q22.3**	**PWP2 periodic tryptophan protein homolog**	**1.92**
**36088_at**	***DSCR2***	AJ006291	**21q22.3**	**Leucine-rich protein**	**1.74**
34414_at	*KIAA0368*	AB002366	9q31.3	mRNA for KIAA0368 gene	1.69
34367_at	*PHGDH*	AF006043	1p12	3-phosphoglycerate dehydrogenase	1.66
**35776_at**	***ITSN1***	AF064243	**21q22.1-q22.2**	**Intersectin 1 (SH3 domain protein)**	**1.56**
**32236_at**	***UBE2G2***	AF032456	**21q22.3**	**Similar to UBC7; ubiquitin conjugating enzyme**	**1.50**
**39348_at**	***HRMT1L1***	X99209	**21q22.3**	**HMT1 hnRNP methyltransferase-like 1**	**1.50**
38942_r_at	*AD024*	W28610	2q24.3	Kinetochore protein Spc25	1.45
40959_at	*PLEKHG3*	AB011171	14q23.3	Pleckstrin homology and rho GEF family	1.38
32738_at	*NDUFS2*	AF050640	1q23	NADH-coenzyme Q reductase	1.36

**Table 3:** Genes over-expressed in late passage +21 fibroblasts passing ANOVA at p < .01. Genes on chromosome 21 are in bold.

**Affy ID**	**Gene name**	**Genbank**	**Chromosome**	**Description**	**Fold change**
**37014_at**	***MX1***	M33882	**21q22.3**	**Myxovirus resistance 1, interferon-inducible p78**	**16.02**
37111_g_at	*PFKFB3*	AB012229	10p14-p15	6-phosphofructo-2-kinase	2.26
32714_s_at	*ACVRL1*	L17075	12q11-q14	activin A receptor type II-like 1	2.01
34157_f_at	*HIST1H2AL*	AI200373	6p22-p21.3	Histone 1, H2al	1.90
844_at	*PPP1R1A*	U48707	12q13.2	Protein phosphatase 1, regulatory subunit 1A	1.82
31559_at	*SLC13A2*	U26209	17p13.2	Sodium-dependent dicarboxylate transporter	1.73
32811_at	*MYO1C*	X98507	17p13	Myosin IC	1.68
32980_f_at	*H2BFL*	AI688098	6p21.3	HISTONE H2B histone family, member L	1.68
35091_at	*NRG2*	AA706226	5q23-q33	Neuregulin 2	1.64
40370_f_at	*HLA-G*	M90683	6p21.3	histocompatibility antigen, class I, G	1.57
32474_at	*PAX7*	X96744	1p36.2-p36.12	Paired box gene 7	1.51

Several noteworthy features were seen in the expression data from the early passage cells. First, genes on chromosome 21 were strikingly over-represented, relative to those on other chromosomes, in the 'high in +21' set. The proportion of chromosome 21 genes was 6/14 (43%) by ANOVA at p < .01, Table 2, and 17/58 (29%) by ANOVA at p < .05 [see Additional file 1]. By comparison, the random expectation based on the number of genes on chromosome 21 divided by the number of genes on all human chromosomes represented on the microarray is only ~1%. Second, no genes on chromosome 21 were present in the 'low in +21' sets. Third, in these early passage cells the magnitude of the observed over-expression for most of the genes in the 'high in +21' subsets was small, generally within the range expected from gene dosage (1.5X) (Table 2 and [see Additional file 1]). These results indicate that the cell cannot fully adjust for the presence of the extra chromosome 21, i.e. at least some genes on this chromosome are not subject to compensatory down-modulation in the trisomic early passage fibroblasts.

### The interferon target gene MX1 is non-linearly over-expressed in late passage fibroblasts with +21

**Figure 2:**
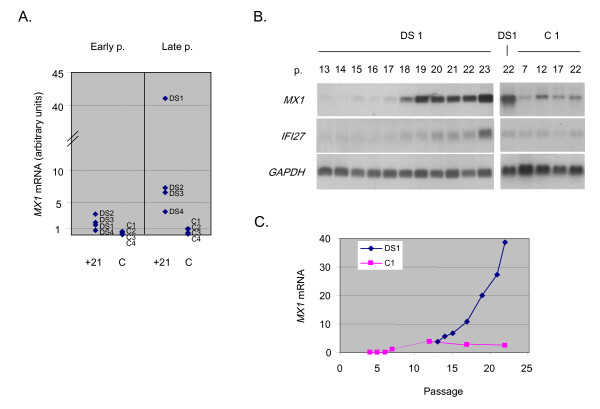
**Over-expression of *MX1 *mRNA in fibroblasts with +21. A, **Scatter plot of the GeneChip data for *MX1*, showing the range of expression of this gene in +21 and control fibroblasts at early and late passage. The data in this figure are from the control and +21 fibroblast lines at the passage numbers and percentage of cells positive for acid beta-galactosidase listed in Table 1. **B, **northern blots showing the over-expression of *MX1 *mRNA, particularly with increasing passage, in a +21 fibroblast line (DS1) compared to a control fibroblast line (C1). Passage number is indicated above each lane. Re-hybridization with a *GAPDH *cDNA probe is shown as a loading control. The blots were stripped and re-hybridized with an *IFI27 *cDNA probe, indicating that this interferon-inducible gene also becomes over-expressed as the cells senesce; but with somewhat less differential expression than *MX1*. Similar northern blotting results were obtained with two other control and two other +21 fibroblast lines (data not shown). **C**, Quantitation of the northern blot signals by Phosphorimaging shows increasing *MX1 *mRNA (normalized to *GAPDH*) as a function of passage number in +21 fibroblasts, with a more modest increase in control fibroblasts.

The expression data in the late passage fibroblasts showed similarities and differences compared to the results in the early passage cells. Only a single chromosome 21 gene passed the 1-way (single parameter, presence or absence of +21) ANOVA test at p < .01 in these senescing cells, and this gene, *MX1*, was over-expressed far in excess of the 1.5-fold change expected from chromosome dosage in the +21 fibroblasts (mean fold increase compared to control fibroblasts = 16, Table 3). In addition to *MX1*, a second closely related chromosome 21-linked gene, *MX2*, passed the less stringent ANOVA at p < .05 [see Additional file 2]. The over-expression of *MX1 *in senescing +21 fibroblasts, with progressively increasing expression with increasing passage number, was verified by northern blotting (Fig. 2b,c). In normal fibroblasts, we observed a small increase in *MX1 *expression at late passages, but these cells never achieved the very high levels of *MX1 *mRNA seen in the +21 lines (Fig. 2a–c). Accordingly, in 2-way ANOVA using both p < .01 and p < .001 criteria, with the 2 parameters of passage (early, late) and genetic characteristic (normal, +21), the *MX1 *gene did not appear among the genes whose mRNA levels were associated with senescence independently of genetic characteristic, but did appear among the genes whose mRNA levels were associated with the presence or absence of +21. The gene list from the 2-way ANOVA with a cut-off of p < .001 for differential mRNA expression as a function of early vs. late passage, independent of genetic characteristic, is provided as a supplemental table [see Additional file 3]. While we will remain focused primarily on the effects of +21 in this report, this list of candidate general senescence-associated genes may be useful for future studies.

**Figure 3:**
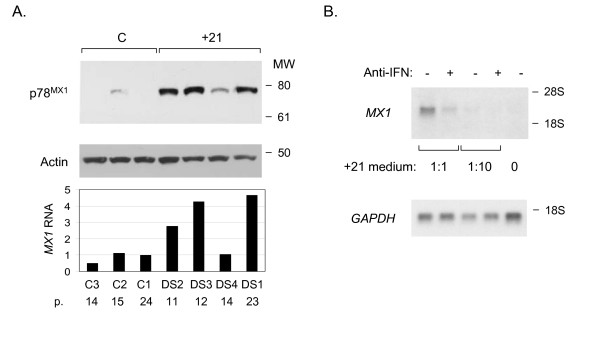
**Validation of p78^MX1 ^over-expression in +21 fibroblasts by western blotting, correlation of p78^MX1 ^protein with *MX1 *mRNA, and induction of *MX1 *mRNA in normal fibroblasts by interferon-beta present in +21 fibroblast-conditioned medium. A, **p78^MX1 ^is over-expressed in +21 fibroblasts, directly correlating with *MX1 *RNA. The top two panels show results from a western blot of proteins from whole-cell lysates of +21 (n = 4) and control (n = 3) fibroblasts at late passage. The blot was re-probed with anti-beta-actin is as a loading control. The bottom panel shows the relative *MX1 *RNA levels of all the samples from the GeneChip data, normalized to the value for the late-passage C1 fibroblasts. P, passage number. The excess of *MX1 *mRNA in these cells leads to the expected excess production of p78^MX1 ^protein. **B, **conditioned medium from fibroblasts with trisomy 21 was added to the medium of control fibroblasts at the indicated proportions, either with our without the addition of anti-interferon beta antibodies. RNA was extracted after 24 hours and analyzed by northern blotting. Addition of anti-interferon almost completely abrogates the induction of *MX1 *mRNA by the +21 conditioned medium.

The correlation coefficient for the level of *MX1 *mRNA (GeneChip data) vs. the percentage of acid beta-galactosidase-positive cells was 0.63 for normal fibroblasts and 0.72 for the +21 fibroblasts. Thus, *MX1 *expression was correlated to cell senescence in both types of fibroblasts, albeit with a stronger positive correlation in +21 compared to control, and with a substantially greater magnitude of the increased expression in the +21 cells. In addition, western blotting of total cellular protein indicated that the excess of *MX1 *mRNA in these cells led to the expected excess production of p78^MX1 ^protein, and we observed a good correlation between *MX1 *mRNA and p78^MX1 ^protein (Fig. 3a).

The *MX1 *gene is a well known target of type-I interferon signaling, as is *MX2 *[16,17]. While the increase in *MX1 *mRNA was the most dramatic effect seen during +21 fibroblast senescence, re-probing of the northern blots with an additional interferon-inducible gene, *IFI27*, revealed a weaker but parallel increase in mRNA expression (Fig. 2a). *IFI27 *showed a 9-fold mean increase in the late-passage +21 fibroblasts in our microarray data, but this gene was screened out by our data analysis protocol due to an insufficient number of Affymetrix presence calls (see Methods). Although *IFI27 *is not on chromosome 21, the receptors for interferon alpha and beta are encoded by genes on chromosome 21. While we did not detect increased mRNA for these genes in our survey, fibroblasts and other cells with +21 are known to express an increased density of cell surface interferon receptors at the protein level, and to be hyper-responsive to signaling by exogenous interferon [18-24]. Gene-gene interaction, namely the presence of an extra gene copy of both an upstream and a downstream component of the interferon-signaling pathway, is a likely explanation for the non-linear over-expression of *MX1 *mRNA and protein in +21 cells. Indeed, in early work the protein product of the *MX1 *gene, p78^MX1^, was found over-expressed in two lines of +21 fibroblasts, compared to control fibroblasts, after these cells were exposed to exogenous interferon [25,26]. Our current data add to this information by showing strong constitutive over-expression of *MX1 *in senescing +21 fibroblasts, and we therefore wished to test whether this increased expression might be maintained by autocrine interferon signaling. Consistent with this hypothesis, conditioned medium from +21 fibroblast cultures induced the expression of *MX1 *mRNA in control fibroblasts, and this induction was attenuated by the addition of anti-interferon-beta antibodies to the cell cultures (Fig. 3b).

### Induction of p78^MX1 ^in lesional tissue of alopecia areata

**Figure 4:**
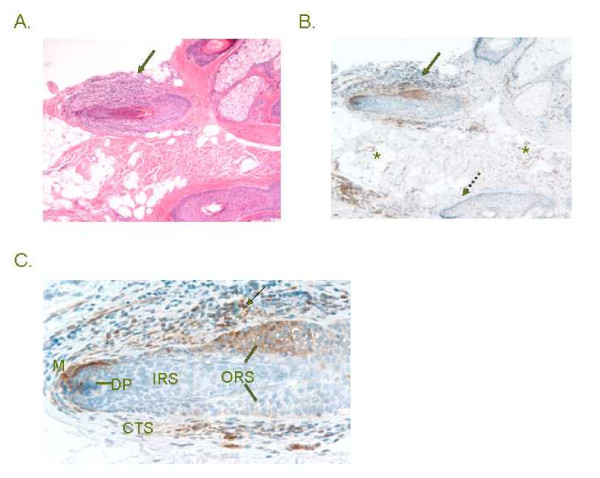
**Activation of p78^MX1 ^in lymphocytes and epithelial cells in a case of alopecia areata. A, **low power field of a scalp biopsy with alopecia areata, showing an inflamed hair follicle (arrow) (H&E). **B, **low power field of staining with anti-p78^MX1 ^showing positive cells in and around the inflamed hair follicle (solid arrow), but not in an uninvolved region of another hair follicle (dashed arrow). Capillary endothelial cells stain throughout the dermis (asterisks). **C, **high power field of staining with anti-p78^MX1^, showing that the p78-positive cells are a subset of lymphocytes (arrow) and outer root sheath epithelial cells (ORS, lines), as well as matrix cells (M) around the dermal papilla (DP). The inner root sheath (IRS) does not stain. A second case of sporadic alopecia areata revealed a similar pattern of staining (data not shown). CTS = connective tissue sheath.

A previous genetic association study linked intronic polymorphisms in the *MX1 *gene to the inflammatory hair loss disorder alopecia areata [27]. This condition has long been recognized to have an increased (up to 90-fold) prevalence in DS [28-31]. We therefore used immunohistochemistry to assess the expression of p78^MX1 ^in scalp biopsies. In control scalp biopsies, p78^MX1 ^expression was restricted to the capillary endothelial cells (data not shown). In both of two cases of sporadic alopecia areata that we examined there was high expression both in endothelial cells and in a subset of the infiltrating lymphocytes and in epithelial cells (outer root sheath cells and matrix cells) of the inflamed hair follicles, with no immunoreactivity in the adjacent uninvolved hair follicles (Fig. 4a–c and data not shown). We did not have access to alopecia areata biopsies from DS, but examining such cases will be desirable in future studies.

### The GART gene is non-linearly over-expressed in +21 mid-gestation fetal hearts

**Figure 5:**
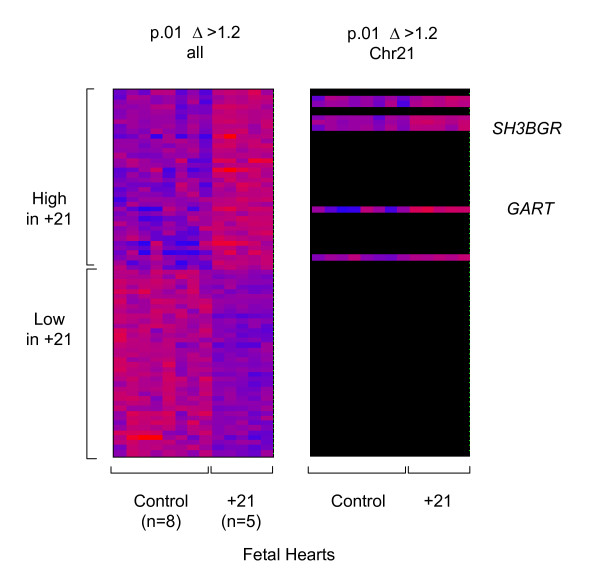
**Differential gene expression in control vs. +21 fetal hearts**. Tissue samples were obtained from fetal hearts at 15 to 23 weeks gestation. Results from supervised hierarchical clustering of the U95A microarray data are shown; with statistical criteria for selecting the genes indicated at the top (ANOVA, see Methods) and the expression values displayed as in Figure 1.

**Figure 6:**
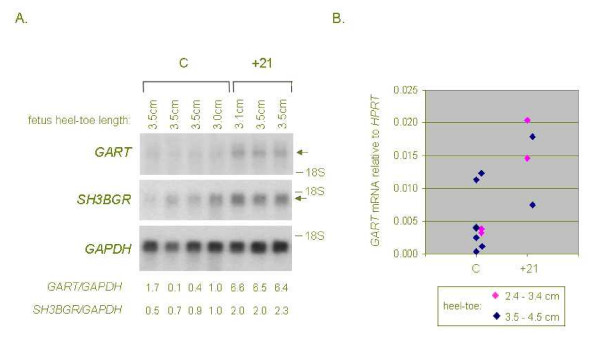
**Validation of strong over-expression of *GART *and moderate over-expression of *SH3BGR *mRNA in mid-gestation fetal hearts with +21 by northern blotting and real-time RT-PCR. A, **northern blot containing total RNA from control and +21 fetal hearts at 17 to 19 weeks gestation (3.0 to 3.5 cm heel-toe length) was hybridized with the indicated cDNA probes, with stripping of the blot between hybridizations. Arrows indicate the specific transcripts, and the dashes show positions of the 18S ribosomal RNA as a size marker. The ratio of *GART *and *SH3BGR *signals to that obtained with the *GAPDH *probe, as measured by Phosphorimaging, is shown below each lane. **B, **real-time RT-PCR analysis of *GART *mRNA in control and +21 fetal hearts. The values for *GART *mRNA were normalized to *HPRT *expression in each sample. Stages of development (heel-toe length) are color-coded.

A similar supervised analysis of microarray data from 5 fetal hearts with +21 compared to 8 control fetal hearts matched for gestational age (mean G.A. in +21 cases = 16.8 weeks; mean G.A. in controls = 18.5 weeks, t-test p = .2) also revealed a systematic over-expression of chromosome 21-linked genes in the +21 cases. Using ANOVA at a cutoff of p < .01, among 37 genes in the 'high in +21' category, 7 (19%) mapped to chromosome 21; while no chromosome 21-linked genes were in the under-expressed category (Fig. 5 and Table 4). Similarly, the statistical analysis of the microarray data for these samples at p < .05 showed 17/110 (15%) of the over-expressed genes mapping to chromosome 21, a proportion much higher than the 1% expected from the overall gene representation on this small chromosome [see Additional file 4]. The chromosome 21 gene with the strongest over-expression in this dataset was *GART*, encoding a metabolic enzyme with three catalytic activities (phosphoribosylglycinamide formyltransferase, phosphoribosylglycinamide synthetase, phosphoribosylaminoimidazole synthetase), which acts at several steps of purine biosynthesis [32]. In addition, several other chromosome 21 genes, including *SH3BGR*, encoding a coiled-coil and SH3 domain-containing protein, were modestly over-expressed on average in the +21 hearts (Table 4 and [see Additional file 4]). *GART *mRNA, while not an abundant transcript, is detectable by northern blotting with prolonged exposures, and we were able to verify the strong (non-linear) over-expression of this gene, and the modest over-expression of *SH3BGR *mRNA, by northern blotting and Phosphorimaging in 3 of the hearts with +21 for which ample RNA remained after the GeneChip experiment, compared to 4 control hearts closely matched for gestational age (Fig. 6a). This result was further confirmed by real-time RT-PCR comparing *GART *mRNA expression (normalized to mRNA from the *HPRT *housekeeping gene) in 4 of the +21 fetal hearts compared to 6 of the control fetal hearts from our GeneChip series (Fig. 6b).

**Table 4:** Genes over-expressed in +21 fetal hearts (ANOVA p < .01). Genes mapping to chromosome 21 are in bold.

**Affy ID**	**Gene Name**	**Genbank**	**Chromosome**	**Description**	**Fold change**
33455_at	*ALDOB*	X02747	9q21.3-q22.2	Aldolase B	4.56
32027_at	*PDZK1*	AF012281	1q21	PDZ domain containing 1	3.99
39151_at	*PAPPA2*	AL031290	1q23-q25	Pregnancy-associated plasma protein	3.47
425_at	*ISG12 IFI27*	X67325	14q32	Interferon, alpha-inducible protein 27	3.47
**38384_at**	***GART***	X54199	**21q22.11**	**Trifunctional metabolic enzyme (see text)**	**3.02**
35692_at	*RIS1*	AL080235	3p21.3	Ras-induced senescence 1	2.63
37202_at	*F2*	J00307	11p11-q12	Prothrombin	2.15
36256_at	*LSAMP*	U41901	3q13.2-q21	Limbic system-associated membrane protein	2.10
36245_at	*HTR2B*	X77307	2q36.3-q37.1	5-hydroxytryptamine (serotonin) receptor 2B	2.05
34316_at	*RPS15A*	W52024	16p	Ribosomal protein S15a	1.96
37871_at	*IAPP AMYLIN*	X68830	12p12.3-p12.1	Islet amyloid polypeptide (IAPP).	1.94
38201_at	*ECA39*	U21551	12pter-q12	Branched chain aminotransferase 1, cytosolic	1.88
33203_s_at	*FREAC-4*	U59831	5q12-q13	Forkhead related activator 4 (FREAC-4)	1.87
39248_at	*AQP3*	N74607	9p13	Aquaporin 3	1.82
37399_at	*AKR1C3*	D17793	10p15-p14	3-alpha hydroxysteroid dehydrogenase, type II	1.81
32486_at	*CKMM*	AC005781	19q13.2-q13.3	Creatine kinase, muscle	1.81
1574_s_at	*IL4*	M13982	5q31.1	Interleukin 4	1.72
39674_r_at	*ECM2*	AB011792	9q22.3	Extracellular matrix protein 2	1.72
**38738_at**	***SUMO3***	X99584	**21q22.3**	**SMT3 suppressor of mif two, homolog 3**	**1.66**
36155_at	*SPOCK2*	D87465	10pter-q25.3	Sparc/osteonectin, proteoglycan (testican) 2	1.63
31623_f_at	*MT1A*	K01383	16q13	metallothionein-I-A	1.61
32250_at	*CFH*	X07523	1q32	Complement factor H	1.61
40587_s_at	*EEF1E1*	AF054186	6p24.3-p25.1	Eukaryotic elongation factor 1 epsilon 1	1.60
**36107_at**	***ATP5J***	AA845575	**21q21.1**	**ATP synthase, H+ transporting, mitochondrial**	**1.55**
201_s_at	*B2M*	S82297	15q21-q22.2	Beta-2-microglobulin	1.54
**39767_at**	***CCT8***	D13627	**21q22.11**	**Chaperonin containing TCP1, subunit 8 (theta)**	**1.53**
37177_at	*CD58*	Y00636	1p13	CD58 antigen, (LFA-3)	1.52
33358_at	*PPM1H*	W29087	12q14.1-q14.2	Protein phosphatase	1.52
**36039_s_at**	***SH3BGR***	X93498	**21q22.3**	**SH3 domain binding glutamic acid-rich protein**	**1.51**
34995_at	*CALCRL*	L76380	2q32.1	Calcitonin receptor-like	1.46
37231_at	*DLG7*	D13633	14q22.3	Discs, large homolog 7 (Drosophila)	1.44
**39005_s_at**	***ZNF294***	AB018257	**21q22.11**	**Zinc finger protein 294**	**1.43**
**879_at**	***MX2***	M30818	**21q22.3**	**Myxovirus (influenza virus) resistance 2**	**1.40**
38066_at	*NQO1*	M81600	16q22.1	NAD(P)H:quinone oxireductase	1.40
34667_at	*NFX1*	U15306	9p13.3	Nuclear transcription factor, X-box binding 1	1.40
33834_at	*CXCL12*	L36033	10q11.1	Chemokine (stromal cell-derived factor 1)	1.39
37264_at	*ZNF131*	U09410	5p12-p11	Zinc finger protein 131	1.35

**Figure 7:**
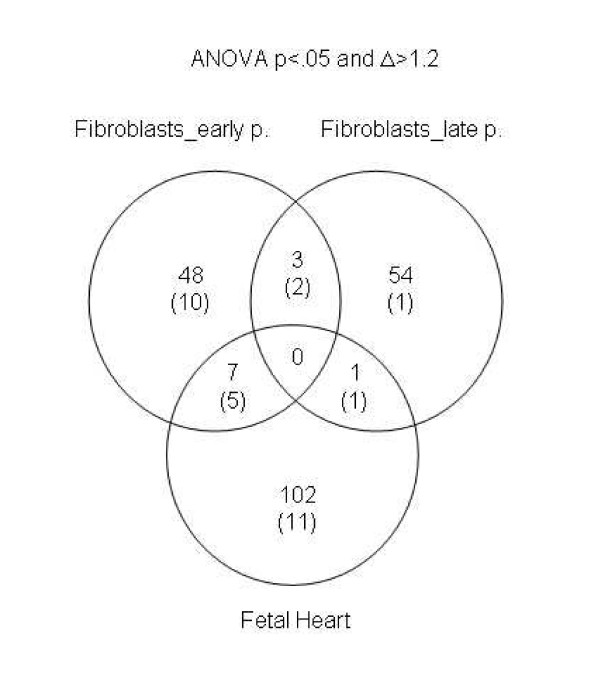
**Venn diagram showing the limited overlap of the over-expressed genes in the three cell types/tissues analyzed in this study**. The total numbers of genes are indicated, with the number of genes mapping to chromosome 21 shown in parentheses.

As seen by comparing Tables 2, 3, 4 and the supplemental tables [see Additional file 1], [see Additional file 2], [see Additional file 4], the genes differentially expressed as a function of +21 in the fetal hearts did not overlap substantially with the genes that were differentially expressed in the early- or late-passage fibroblasts. The two exceptions were *SH3BGR*, which was modestly (i.e. linearly) over-expressed in the hearts and early passage fibroblasts with +21, and *MX2*, which was modestly over-expressed in both the hearts and the late passage fibroblasts. The limited overlaps between the gene lists from the supplemental tables are highlighted by the Venn diagram in Figure 7. Among the few genes that appear in the regions of overlap in this diagram, a strikingly large proportion map to chromosome 21.

## Discussion

A number of screens for altered gene expression in DS cells and tissues, and in the segmental trisomy 16 mouse models of DS, have been published over the past several years. Analysis of mRNAs in brain is clearly of major interest, but several prominent aspects of the DS phenotype involve non-CNS tissues. Our analysis of gene expression in fibroblasts and fetal hearts with trisomy 21 leads us to a series of conclusions. First, the extra chromosome 21 does not cause widespread strong dysregulation of gene expression. Second, genes on chromosome 21 are in fact disproportionately over-expressed in the +21 cells compared with genes on other chromosomes. So while regulatory feedback mechanisms are likely to exist, the data make it clear that fibroblasts and fetal heart cells do not completely compensate for this chromosomal aneuploidy. A similar conclusion has been reached by other groups studying the segmental trisomy 16 mouse [6,7,33]. Third, small subsets of genes on other chromosomes are either over- or under-expressed in the setting of +21. We have focused here on the over-expressed genes, but genes that are down-modulated may also be biologically relevant, and these genes are listed in the supplemental tables [see Additional file 1], [see Additional file 2], [see Additional file 4]. Fourth, the sets of genes differentially expressed as a function of the trisomy differ in different cell types. We found that the interferon-inducible *MX1 *gene is strongly (non-linearly) over-expressed in senescing +21 fibroblasts, and that the *GART *gene, encoding a multifunctional enzyme in de novo purine biosynthesis, is non-linearly over-expressed in mid-gestation fetal hearts with +21. For *MX1*, non-linear over-expression can be explained at least in part by the existence of more than one interferon pathway component encoded on chromosome 21. We have also shown evidence for induction of the *MX1 *gene product, p78^MX1^, in lesional hair follicle tissue of a sporadic case of alopecia areata, an autoimmune disorder that has an elevated frequency in DS. In terms of these genes in the prior literature, *Gart *was found slightly up-regulated in heart, kidney, and muscle of segmental trisomy 16 mice at day 30 by 1.54, 1.68, and 1.83, respectively [8,11], and in the expression profiling studies by Kahlem et al. and Lyle et al., the murine *Mx1 *and *Mx2 *genes appeared among the limited set of chromosome 16-linked genes (13 genes) found to have >2-fold over-expression in several adult tissues at postnatal day 10 and at 11 months in the segmental trisomy 16 mice [8,11].

*MX1 *encodes a large GTPase that forms organized multimeric structures in the cytoplasm. This protein is a well known interferon target gene, and studies both in cell culture and in vivo have documented that it is an essential component of the antiviral response. How this antiviral activity is exerted is not yet clear, and it appears that multiple steps in the viral life cycle are inhibited by p78^MX1 ^[34]. But p78^MX1 ^may also function outside of anti-viral defense. A link between *MX1 *and sporadic alopecia areata has been raised by a reasonably large genetic association study, including 165 alopecia areata cases and 510 controls, which showed that the +9959 intronic polymorphism conveyed a 1.79-fold increase in risk [27]. Since alopecia areata is about 90 times more frequent in DS than in the general population, our findings that *MX1 *is non-linearly over-expressed in +21 cells, and that this gene is activated in inflamed hair follicles in alopecia areata, add credence to *MX1 *as an alopecia areata susceptibility locus. Clinical studies using high-dose interferons to treat hepatitis and other conditions have reported alopecia as a common side effect, so intense and prolonged interferon signaling is clearly sufficient to produce alopecia [35-40]. Additional work will be needed to determine whether p78^MX1 ^is playing an effector role in the arrest of the hair cycle in alopecia areata.

Our observation that *MX1 *mRNA is modestly upregulated in normal fibroblasts during senescence is consistent with a report by Yoon et al., who used a cDNA microarray to screen for genes upregulated during replicative senescence, in a panel of 3 normal human fibroblast lines [41]. Based on our finding that *MX1 *mRNA and p78^MX1 ^protein are much more strongly induced as +21 fibroblasts enter replicative senescence, it will be interesting to study the role of this gene, and of the interferon pathway more generally, in the process of premature aging and cell senescence. Various aspects of premature aging have been studied as phenotypes in DS [42,43], and premature skin wrinkling is accepted as a genuine component of this syndrome [44]. However, whether +21 dermal fibroblasts really show reduced replicative potential has been controversial, and it has been easier to document deficiencies in DNA repair than in replicative lifespan in +21 compared to control fibroblast lines [45,46]. Relevant to this topic, it has been reported that epigenetic silencing of multiple interferon-response genes, including *MX1*, occurs with cellular immortalization in tissue culture [47], suggesting that there is in fact a strong selective pressure to lose this pathway in order for the cells to escape senescence.

Cardiac defects, notably atrioventricular defects due to anomalous development of structures derived from the endocardial cushions, are a major adverse consequence of +21 [48-51], but the basis for abnormal heart development in this condition has remained unclear. Molecular candidates include proteins expressed in cardiac development that are encoded by chromosome 21 genes, such as the adhesion protein gene *DSCAM*, collagen VI (*COL6AI *and *COL6A2 *genes), the calcineurin pathway modulator *DSCR1*, the *SH3BGR *gene, and others [52-61]. Of these genes, *SH3BGR *was over-expressed on average in the +21 fetal hearts in our series, while *DSCAM *and the collagen VI genes were not (*DSCR1 *was modestly over-expressed, [see Additional file 4]). These negative findings do not exclude these genes as important loci: for example, we sampled large pieces of heart tissue, but *COL6AI *mRNA is known to be abundant only in the normal endocardium and endocardial cushions [57,60,62] and its over-expression in +21 fetal hearts might only be evident after tissue microdissection. In fact, while this paper was under review, Mao et al. reported *COL6A1 *mRNA elevated 1.4-fold in comparing 3 fetal hearts with +21 to 3 control fetal hearts, all dissected to enrich for the area of the atrio-ventricular valves and septum, using a microarray method similar to ours [63]. It can also be argued that minimal over-expression over an extended time in development, or strong over-expression in a narrow time window, could have significant biological effects. Nonetheless, our positive data highlight another gene, *GART*, which might contribute via its strong over-expression to cardiac defects in DS. Purine biosynthesis can affect intracellular nucleotide pools as well as purinergic signaling between cells and it will be interesting to explore the functional role of *GART *in heart development. Why this gene should be non-linearly over-expressed is not yet clear, but a previous study using western blotting documented the relative over-expression of the GARS-AIRS-GART protein in post-natal cerebellum in human DS and, interestingly, found that this over-expression was due to the failure of a normal program of *GART *silencing during cerebellar maturation [64]. Examination of additional cases over a wider range of gestational age will be needed to ask whether the over-expression of *GART *in +21 fetal hearts also reflects aberrant developmental regulation.

Gene expression measurements are only circumstantial evidence, and transgenic experiments in mice [53,55], including the recently reported whole human chromosome 21 transfer model [65], will be necessary to sort through each of these candidate genes for their functional relevance. Cardiac defects associated with +21 have been reported in mice engineered to carry human chromosome 21 [65-67], and this system may prove useful for further dissecting the genetics. The other important approach, genetic mapping using partial trisomies in humans, has led to evidence that the *DSCAM *gene may be in a critical region for Tetralogy of Fallot [52,54]. *GART *(34 Mb on the physical map) is located substantially centromeric to *DSCAM *(41 Mb), outside this region.

From a more general viewpoint, our data and those from other laboratories cited above clearly show that human and mouse cells and tissues do not effectively compensate for simple chromosomal aneuploidies, and that this lack of compensation manifests as abnormal steady-state mRNA levels from different sets of genes in different cell types and tissues. For some genes the perturbations in mRNA expression resulting from these simple chromosome gains are non-linear, and it may be a general principle that this phenomenon can be explained by gene-gene interactions, as exemplified here by *MX1 *and other genes in the interferon pathway.

## Conclusion

Our results indicate over-expression of chromosome 21 genes in human cells and tissues with +21, and highlight different subsets of chromosome 21 genes as selectively over-expressed in different cell types. Our data further indicate that for some chromosome 21 genes this over-expression is non-linear (>1.5X). Based on the observed non-linear over-expression of *MX1 *mRNA and p78^MX1 ^protein in senescent fibroblasts with +21, and the over-expression of p78^MX1 ^in inflamed hair follicles of alopecia areata, hyperactive interferon signaling is a candidate pathway for cell senescence and autoimmune disorders in DS. Additionally, the strong over-expression of the *GART *gene that we observe in fetal hearts with +21 suggests that abnormal purine metabolism should be investigated for a potential role in the cardiac defects associated with DS.

## Abbreviations

**DS **– Down syndrome, **+21 **– trisomy 21.

## Competing interests

The author(s) declare that they have no competing interests.

## Authors' contributions

Chi-Ming Li carried out the experiments shown in all of the figures. Meirong Guo participated in carrying out the experiments in figures 1, 2, 3. Martha Salas participated in carrying out the experiments in Figures 4, 5, 6. Nicole Schupf, Wayne Silverman and Warren Zigman participated in the statistical analyses and in writing the manuscript, as well as in obtaining grant funding. Sameera Husain made the histopathological diagnosis in the cases of alopecia areata. Dorothy Warburton directs the cytogenetics laboratory and participated in writing the manuscript. Harshwardhan Thaker directs the Tissue Bank and participated in writing the manuscript. Benjamin Tycko directed the experimental design of this study and participated in the statistical analyses, writing the manuscript and obtaining grant funding. All authors approved and read the final manuscript.

## Pre-publication history

The pre-publication history for this paper can be accessed here:



## Supplementary Material

Additional File 1 Genes over- or under-expressed in early passage fibroblasts with +21. ANOVA, p < .05. Click here for file

Additional File 2 Genes over- or under-expressed in late passage fibroblasts with +21. ANOVA, p < .05. Click here for file

Additional File 3 Genes over- or under-expressed in late passage compared to early passage fibroblasts, independently of +21 (2-way ANOVA, p < .001.). Click here for file

Additional File 4 Genes over- or under-expressed in fetal hearts with +21. ANOVA, p < .05. Click here for file
