# Supplementary material for: Cell type-specific over-expression of chromosome 21 genes in fibroblasts and fetal hearts with trisomy 21
Source: BMC Med Genet. 2006 Mar 15;7:24. doi: 10.1186/1471-2350-7-24 (PMC1435874; doi:10.1186/1471-2350-7-24)
Supplement: Additional File 1 — Genes over- or under-expressed in early passage fibroblasts with +21. ANOVA, p < .05. [file 1471-2350-7-24-S1.pdf]

Supplemental Table 1. Genes over- or under-expressed in early passage fibroblasts with +21. ANOVA, p<.05.

Affy ID	Synonyms	Symbol	Genbank	Description	Chromosome	Fold difference
41585_at	KIAA0746	KIAA0746	AB018289	KIAA0746 protein	4p15.2	5.21
36873_at	VLDLR	VLDLR	D16532	Very low density lipoprotein receptor	9p24	3.63
1616_at	FGF-9	FGF9	D14838	Fibroblast growth factor 9	13q11-q12	3.30
**36040_at**	**SH3BGR**	**SH3BGR**	**AI337192**	**SH3 domain binding glutamic acid-rich protein**	**21q22.3**	**3.10**
34348_at	SPINT2	SPINT2	U78095	Serine protease inhibitor, Kunitz type, 2	19q13.1	2.93
**37014_at**	**MX1**	**MX1**	**M33882**	**Myxovirus (influenza virus) resistance 1, interferon-inducible protein p78**	**21q22.3**	**2.70**
1767_s_at	TGF-beta 3	TGFB3	X14885	Transforming growth factor-beta 3	14q24	2.67
**35714_at**	**PDXK**	**PDXK**	**U89606**	**Pyridoxal (pyridoxine, vitamin B6) kinase**	**21q22.3**	**2.43**
**38384_at**	**GART**	**GART**	**X54199**	**Phosphoribosylglycinamide formyltransferase, phosphoribosylglycinamide synthetase, phosphoribosylaminoimidazole synthetase**	**21q22.11**	**2.27**
**38474_at**	**CBS**	**CBS**	**L00972**	**Cystathionine-beta-synthase**	**21q22.3**	**2.24**
34542_at	gapdh-2	GAPDS	AJ005371	Glyceraldehyde-3-phosphate dehydrogenase, spermatogenic	19q13.1	2.21
40009_at	FMR2	FMR2	U48436	Fragile X mental retardation 2	Xq28	2.19
**37555_at**	**PWP2**	**PWP2H**	**X95263**	**periodic tryptophan protein homolog**	**21q22.3**	**2.01**
35305_at	XPNPEPL	XPNPEP1	X95762	X-prolyl aminopeptidase 1, soluble	10q25.3	2.00
39366_at	PPP1R3C	PPP1R3C	N36638	Protein phosphatase 1, regulatory (inhibitor) subunit 3C	10q23-q24	1.99
31840_at	IDE	IDE	M21188	Insulin-degrading enzyme	10q23-q25	1.98
39420_at	TLS/CHOP	DDIT3	S62138	DNA-damage-inducible transcript 3	12q13.1-q13.2	1.81
39219_at	CEBPG	CEBPG	U20240	CCAAT/enhancer binding protein (C/EBP), gamma	19q13.11	1.79
**34994_at**	**N143**	**WDR9**	**AJ002572**	**Chromosome 21 open reading frame 107**	**21q22.2**	**1.78**
**36088_at**	**c21-lrp**	**DSCR2**	**AJ006291**	**Down syndrome critical region gene 2**	**21q22.3**	**1.74**
37188_at	PCK2	PCK2	X92720	Phosphoenolpyruvate carboxykinase 2 (mitochondrial)	14q11.2	1.73
34849_at	serS	SARS	X91257	Seryl-tRNA synthetase	1p13.3-p13.1	1.73
37032_at	NNMT	NNMT	U08021	Nicotinamide N-methyltransferase	11q23.1	1.71
**39767_at**	**KIAA0002**	**CCT8**	**D13627**	**Chaperonin containing TCP1, subunit 8 (theta)**	**21q22.11**	**1.68**
34367_at	PHGDH	PHGDH	AF006043	A10; 3-phosphoglycerate dehydrogenase	1p12	1.66
41252_s_at	PLEKHB2		W28614	Pleckstrin homology domain containing, family B (evectins) member 2	2q21.1	1.66
38977_at	YARS	YARS	U89436	Tyrosyl-tRNA synthetase	1p35.1	1.64
**31863_at**	**KIAA0179**	**KIAA0179**	**D80001**	**KIAA0179**	**21q22.3**	**1.60**
40782_at	SDR1	SDR1	AF061741	Dehydrogenase/reductase (SDR family) member 3	1p36.1	1.57
35844_at	SDC4; SYNI	SDC4	D79206	Ryudocan core protein	20q12	1.57
**32236_at**	**UBE2G2**	**UBE2G2**	**AF032456**	**Ubiquitin conjugating enzyme G2**	**21q22.3**	**1.56**
**35776_at**	**ITSN1**	**ITSN1**	**AF064243**	**Intersectin 1 (SH3 domain protein)**	**21q22.1-q22.2**	**1.56**
34414_at	KIAA0368	KIAA0368	AB002366	KIAA0368	9q31.3	1.54
**39348_at**	**HRMT1L1**	**HRMT1L1**	**X99209**	**HMT1 hnRNP methyltransferase-like 1**	**21q22.3**	**1.53**
**35816_at**	**cystatin B**	**CSTB**	**U46692**	**EPM1 disease gene; protease inhibitor; cystatin B**	**21q22.3**	**1.53**
41509_at	HSPA9B	HSPA9B	L11066	Heat shock 70kDa protein 9B (mortalin-2)	5q31.1	1.53
35635_at	LOC92249	LOC92249	AL080202	Hypothetical protein LOC92249	Xq11.2	1.52
37243_at	GUCY1B3	GUCY1B3	X66533	Guanylate cyclase 1, soluble, beta 3	4q31.3-q33	1.51
**40116_at**	**PFKL**	**PFKL**	**X15573**	**Phosphofructokinase, liver**	**21q22.3**	**1.51**
41246_at	PN1	SERPINE2	AI743134	Serine proteinase inhibitor; Proteinase nexin 1	2q33-q35	1.48
32229_at	EIF4EL3	EIF4EL3	AF038957	Eukaryotic translation initiation factor 4E-like 3	2q37.1	1.47
32706_at	HIRA	HIRA	X89887	HIR histone cell cycle regulation defective homolog A	22q11.21	1.46
41250_at	JTV-1	JTV1	U24169	JTV1 gene	7p22	1.46
34709_r_at	STAG2	STAG2	Z75331	Nuclear protein SA-2.	Xq25	1.46
38942_r_at	AD024	SPBC25	W28610	Kinetochore protein Spc25	2q24.3	1.45
31497_at	GAGE1	GAGE1	U19142	G antigen 8	Xp11.4-p11.2	1.45
34378_at	ADFP	ADFP	X97324	Adipose differentiation-related protein	9p22.1	1.43
35223_at	AIBP63	HPS5	AB023234	Hermansky-Pudlak 5, integrin binding protein	11p14	1.41
**39003_at**	**PTTG1IP**	**PTTG1IP**	**Z50022**	**Pituitary tumor-transforming 1 interacting protein**	**21q22.3**	**1.40**
37825_at	GK2	GALK2	M84443	Galactokinase 2	15q21.1	1.40
41060_at	CCNE1	CCNE1	M74093	Cyclin E1	19q12	1.40
40959_at	KIAA0599	PLEKHG3	AB011171	Pleckstrin homology domain containing, family G	14q23.3	1.38
39800_s_at	HAX1	HAX1	U68566	HS1 binding protein	1q21.3	1.37
36115_at	clk3	CLK3	L29217	CDC-like kinase 3	15q24	1.36
32738_at	NDUFS2	NDUFS2	AF050640	NADH dehydrogenase (ubiquinone) Fe-S protein 2	1q23	1.36
**36517_at**	**U2AF1**	**U2AF1**	**M96982**	**U2(RNU2) small nuclear RNA auxiliary factor 1**	**21q22.3**	**1.35**
37297_at	LOC84549	LOC84549	AL049422	RNA binding protein 13	8p12	1.35
40882_at	iCE	CES2	Y09616	Carboxylesterase 2 (intestine, liver)	16q22.1	1.32

Affy ID	Synonyms	Symbol	Genbank	Description	Chromosome	Fold difference
36083_at	TSPAN31	SAS	U01160	Sarcoma amplified sequence	12q13.3	0.74
32856_at	KIAA0819	MICAL3	AB020626	Calponin and LIM domain containing 3	22q11.21	0.73
38670_at	RNF114	ZNF313	AL031685	Zinc finger protein 313	20q13.13	0.71
38978_at	PAIP1	PAIP1	AF013758	Poly(A) binding protein interacting protein 1	5p12	0.70
33288_i_at	FPM315	ZNF263	D88827	Zinc finger protein 263	16p13.3	0.69
35363_at	P72	DDX17	AL080113	DEAD (Asp-Glu-Ala-Asp) box polypeptide 17	22q13.1	0.69
34355_at	MeCP2	MECP2	AJ132917	Methyl CpG binding protein 2 (Rett syndrome)	Xq28	0.69
33749_at	P53TG1	TP53AP1	AB007455	TP53 activated protein 1	7q21.1	0.68
34912_at	DRP-1	DAPK2	AF052941	Death-associated protein kinase 2	15q22.31	0.68
34815_at	TNRC12	EP400	U80743	E1A binding protein p400	12q24.33	0.66
41049_at	HIRS-1	IRS1	S62539	Insulin receptor substrate 1	2q36	0.64
851_s_at	HIRS-1	IRS1	S62539	Insulin receptor substrate 1	2q36	0.62
38982_at	RAP1	TERF2IP	W28865	Telomeric repeat binding factor 2, interacting protein	16q23.1	0.62
1461_at	MAD3	NFKBIA	M69043	Nuclear factor of kappa light polypeptide gene enhancer in B-cells inhibitor, alpha	14q13	0.62
1328_at	RNF84	TRAF5	U69108	TNF receptor-associated factor 5	1q32	0.61
37221_at	PRKAR2	PRKAR2B	M31158	Protein kinase, cAMP-dependent, regulatory, type II, beta	7q22	0.61
33002_at	NCKbeta	NCK2	AF047487	NCK adaptor protein 2	2q12	0.60
38902_r_at	CRE-BP1	ATF2	X15875	Activating transcription factor 2	2q32	0.60
36544_at	ARFL3	ARL3	AF038193	ADP-ribosylation factor-like 3	10q23.3	0.60
32506_at	KIAA1108	TBC1D1	AB029031	TBC1 (tre-2/USP6, BUB2, cdc16) domain family, member 1	4p14	0.59
1325_at	MADH1	SMAD1	U59423	SMAD, mothers against DPP homolog 1	4q31	0.58
40814_at	IDS	IDS	L40586	Iduronate 2-sulfatase (Hunter syndrome)	Xq28	0.57
599_at	HLX1	HLX1	M60721	H2.0-like homeo box 1	1q41-q42.1	0.57
33041_at	CX45	GJA7	U03493	Connexin 45, gap junctions protein	17q21.31	0.51
40308_at	KIAA1240	KIAA1240	AI830496	KIAA1240 protein	2p24.1-p23.3	0.50
38066_at	NQO1	NQO1	M81600	NAD(P)H:quinone oxireductase	16q22.1	0.49
38876_at	DKFZp564L	ASB9	AL080091	Ankyrin repeat and SOCS box-containing 9	X	0.44
37111_g_at	PFK2	PFKFB3	AB012229	Fructose-6-phosphate,2-kinase/fructose-2, 6-bisphosphatase	10p14-p15	0.43
1935_at	MDMX	MDM4	AF007111	Transformed 3T3 cell double minute 4	1q32	0.42
36655_at	X104	TJP2	L27476	Tight junction protein 2 (zona occludens 2)	9q13-q21	0.42
1169_at	ME6	PCDHGB7	D88799	Protocadherin gamma subfamily C, 3	5q31	0.41
40303_at	TFAP2C	TFAP2C	U85658	Transcription factor AP-2 gamma	20q13.2	0.38
39247_at	ABCC6	ABCC6	U66689	ATP-binding cassette, sub-family C (CFTR/MRP), member 6	16p13.1	0.33
1788_s_at	MKP-2	DUSP4	U48807	Dual specificity phosphatase 4	8p12-p11	0.32
41354_at	STC	STC1	U25997	Stanniocalcin 1	8p21-p11.2	0.20
