# Supplementary material for: Cell type-specific over-expression of chromosome 21 genes in fibroblasts and fetal hearts with trisomy 21
Source: BMC Med Genet. 2006 Mar 15;7:24. doi: 10.1186/1471-2350-7-24 (PMC1435874; doi:10.1186/1471-2350-7-24)
Supplement: Additional File 2 — Genes over- or under-expressed in late passage fibroblasts with +21. ANOVA, p < .05. [file 1471-2350-7-24-S2.pdf]

Supplemental Table 2. Genes over- or under-expressed in late passage fibroblasts with +21. ANOVA, p<.05.

Affy ID	Synonyms	Symbol	Genbank	Description	Chromosome	fold difference
**37014_at**	**MX1**	**MX1**	**M33882**	**Myxovirus (influenza virus) resistance 1, interferon-inducible protein p78**	**21q22.3**	**16.02**
**879_at**	**MX2**	**MX2**	**M30818**	**Myxovirus (influenza virus) resistance 2**	**21q22.3**	**4.10**
35717_at	KIAA0822	ABCA8	AB020629	ATP-binding cassette, sub-family A (ABC1), member 8	17q24	4.05
40698_at	AICL	CLEC2B	X96719	C-type (calcium dependent, carbohydrate-recognition domain) lectin, superfamily member 2 (activation-induced)	12p13-p12	3.87
846_s_at	Bak	BAK1	U16811	BCL2-antagonist/killer 1	6p21.3	3.85
32387_at	LYPLA3	LYPLA3	AB017494	Lysosomal phospholipase A2	16q22.1	3.30
40850_at	FKBP38	FKBP8	L37033	FK506 binding protein 8, 38kDa	19p12	3.19
1616_at	FGF-9	FGF9	D14838	Fibroblast growth factor 9 (glia-activating factor)	13q11-q12	2.64
34742_at	bcl-xL	BCL2L1	Z23115	BCL2-like 1	20q11.21	2.47
1834_at	GPR	GPR	D38449	Putative G protein coupled receptor	15q14-q15.1	2.45
35017_f_at	CDA12; D6S203	HLA-J	M80469	MHC class I HLA-J	6p21.31	2.43
**37555_at**	**PWP2**	**PWP2H**	**X95263**	**PWP2 periodic tryptophan protein homolog**	**21q22.3**	**2.39**
37111_g_at	PFK2	PFKFB3	AB012229	Fructose-6-phosphate,2-kinase/fructose-2, 6-bisphosphatase	10p14-p15	2.26
867_s_at	THBS1	THBS1	U12471	Thrombospondin 1	15q15	2.18
36530_g_at	MGC2650	MGC2650	AI885381	Hypothetical protein MGC2650	19q13.32	2.17
922_at	PPP2R1A	PPP2R1A	J02902	Protein phosphatase 2A regulatory subunit alpha-isotype	19q13.41	2.14
1615_at	bcl-xL	BCL2L1	Z23115	BCL2-like 1	20q11.21	2.10
33650_at	DDX27	DDX27	W25911	DEAD box polypeptide 27	20q13.13	2.06
35284_f_at	FLJ10738	C14orf114	W28620	Chromosome 14 open reading frame 114	14q24.1	2.03
32714_s_at	ACVRL1	ACVRL1	L17075	Activin A receptor type II-like 1	12q11-q14	2.01
**31927_s_at**	**KNP-I**	**C21orf33**	**D86062**	**Chromosome 21 open reading frame 33**	**21q22.3**	**1.96**
115_at	THBS1	THBS1	X14787	Thrombospondin 1	15q15	1.95
35791_at	MPDU1	MPDU1	AF038961	Mannose-P-dolichol utilization defect 1	17p13.1-p12	1.92
37965_at	PARVB	PARVB	AA181053	Parvin, beta	22q13.2-q13.33	1.90
34157_f_at	H2AF1	HIST1H2AL	AI200373	Histone 1, H2al	6p22-p21.3	1.90
612_s_at	CNP	CNP	M19650	2',3'-cyclic nucleotide 3' phosphodiesterase	17q21	1.89
36841_at	HNAACTE	PTE1	AA811338	Peroxisomal acyl-CoA thioesterase	20q12-q13.1	1.85
33842_at	FLJ11560	KIAA1539	AC004472	KIAA1539; hypothetical protein LOC80256	9p13.3	1.82
844_at	PPP1R1A	PPP1R1A	U48707	Protein phosphatase 1, regulatory (inhibitor) subunit 1A	12q13.2	1.82
1212_at	GSTZ-1	GSTZ1	U86529	Glutathione transferase zeta 1 (maleylacetoacetate isomerase)	14q24.3	1.81
38434_at	AAMP	AAMP	M95627	Angio-associated, migratory cell protein	2q35	1.76
32420_at	GPR6	GPR6	U18549	Putative GPR6 G protein-coupled receptor, ligand unknown	6q21	1.76
38819_at	CCK4	PTK7	U33635	PTK7 protein tyrosine kinase 7	6p21.1	1.75
33758_f_at	PSG13	PSG11	U25988	Pregnancy specific beta-1-glycoprotein 11	19q13.2	1.73
31559_at	NADC1	SLC13A2	U26209	Solute carrier family 13 (sodium-dependent dicarboxylate transporter), member 2	17p13.2	1.73
33214_at	RPMS12	MRPS12	Y11681	Ribosomal protein, mitochondrial, S12	19q13.1-q13.2	1.72
33011_at	NTR2	NTSR2	Y10148	Neurotensin receptor 2	2p25.1	1.69
32980_f_at	HIST1H2BC	H2BFL	AI688098	Histone 1, H2bc	6p21.3	1.68
32811_at	MYO1E	MYO1C	X98507	Myosin-I beta; Myosin IC	17p13	1.68
35598_at	HIST1H3E	H3FD	AA904505	Histone 1, H3e	6p21.3	1.66
35091_at	Don-1	NRG2	AA706226	Neuregulin 2	5q23-q33	1.64
37421_f_at	HLAF	HLA-F	AL022723	Major histocompatibility complex, class I, F	6p21.3	1.64
31523_f_at	H2B/h	H2BFH	Z80780	H2B/h gene.	6p21.3	1.63
1751_g_at			AD000092	Chromosome 19p13.2 cosmids R31240, R30272 and R28549 containing the EKLF, GCDH, CRTC, and RAD23A genes	19p13.2	1.60
40336_at	ADXR	FDXR	J03826	Ferredoxin reductase	17q24-q25	1.60
32866_at	KIAA0605	ADAMTSL2	AB011177	KIAA0605 gene product; ADAMTS-like 2	9q34.2	1.59
40370_f_at	MHC-G	HLA-G	M90683	HLA-G histocompatibility antigen, class I, G	6p21.3	1.57
39640_at	GFAT2	GFPT2	AB016789	Glutamine-fructose-6-phosphate transaminase 2	5q34-q35	1.56
40649_at	NEC1	PCSK1	X64810	Proprotein convertase subtilisin/kexin type 1	5q15-q21	1.54
37490_at	AE3	SLC4A3	L27213	Solute carrier family 4, anion exchanger, member 3	2q36	1.54
34999_at	HVEC	PVRL1	AF060231	Poliovirus receptor-related 1 (herpesvirus entry mediator C; nectin)	11q23	1.54
32474_at	HUP1	PAX7	X96744	Paired box gene 7	1p36.2	1.51
32061_at	FLJ10871	FLJ10871	U96629	FLJ10871 protein	16	1.50
36731_g_at	MRP7	ABCC10	U66684	ATP-binding cassette, sub-family C (CFTR/MRP), member 10	6p21.1	1.43
40369_f_at	MHC-G	HLA-G	AL022723	HLA-G histocompatibility antigen, class I, G	6p21.3	1.43
34093_at			AI829701	IgH chain VDJC region	4	1.43
32151_at	Fug1	RANGAP1	X82260	Ran GTPase activating protein 1	22q13.2	1.42
34260_at	KIAA0683	KIAA0683	AB014583	KIAA0683 gene product	16p13.3	1.35
32082_at	SENP5	SENP5	AL049229	SUMO1/sentrin specific protease 5	3q29	0.73
33828_at	BAF57	SMARCE1	AF035262	SWI/SNF related, matrix associated, actin dependent regulator of chromatin, subfamily e, member 1 BAF57	17q21.2	0.72
35325_at	FBP	RAB14	AF052113	RAB14, member RAS oncogene family	9q32-q34.1	0.72
39555_at	ING1L	ING2	AB012853	Inhibitor of growth family, member 2; Inhibitor of growth family, member 1-like	4q35.1	0.71
38923_at	FSG1	FRG1	L76159	FSHD region gene 1	4q35	0.70

Affy ID	Synonyms	Symbol	Genbank	Description	Chromosome	fold difference
38892_at	DKFZp686P025	KIAA0240	D87077	KIAA0240	6p21.1	0.70
41183_at	CSTF3	CSTF3	U15782	Cleavage stimulation factor, 3' pre-RNA, subunit 3, 77kDa	11p23	0.69
33346_r_at	TUBG	TUBG1	M61764	Tubulin, gamma 1	17q21	0.69
34372_at	KIAA0312	UREB1	AB002310	Upstream regulatory element binding protein 1	Xp11.22	0.69
32218_at	DIPP1	NUDT3	AF034176	Nudix (nucleoside diphosphate linked moiety X)-type motif 3	6p21.2	0.69
41434_at	FLJ12552	USP46	AF070557	Ubiquitin specific protease 46	4q12	0.68
32585_at	EPB41L2	EPB41L2	AF027299	Erythrocyte membrane protein band 4.1-like 2	6p23	0.68
903_at	PP2A	PPP2R5A	L42373	Protein phosphatase 2, regulatory subunit B (B56), alpha isoform	1q32.2-q32.3	0.67
39707_at	KIAA0647	MTMR4	AB014547	Myotubularin related protein 4	17q22-q23	0.67
40431_at	KIAA0431	ASCIZ	AB007891	ATM/ATR-Substrate Chk2-Interacting Zn2+-finger protein	16q23.2	0.67
32853_at	KIAA0719	TOMM70A	AB018262	Translocase of outer mitochondrial membrane 70 homolog A	3q12.2	0.67
40822_at	NF-AT4c	NFATC3	L41067	Nuclear factor of activated T-cells, cytoplasmic, calcineurin-dependent 3	16q22.2	0.67
40455_at	KIAA0830	KIAA0830	AB020637	KIAA0830 protein	11q21	0.66
840_at	ZNF220	MYST3	U47742	MYST histone acetyltransferase (monocytic leukemia) 3	8p11	0.65
35777_at	SNURF	RNF4	AB000468	Ring finger protein 4	4p16.3	0.64
41153_f_at	CTNNA1	CTNNA1	AF102803	Alpha E-catenin (CTNNA1) gene	5q31	0.64
41821_at	CDC2L	CDC2L5	AA203246	Cell division cycle 2-like 5 (cholinesterase-related cell division controller)	7p13	0.64
37735_at	GNG10	GNG10	U31383	Guanine nucleotide binding protein (G protein), gamma 10	9q31.3	0.64
40805_at	KIAA0440	SIPA1L1	AB007900	Signal-induced proliferation-associated 1 like 1	14q24.2	0.64
40472_at	FLJ10257	LOC254531	AF007155	PISC domain containing hypothetical protein	15q14	0.63
38816_at	AZU-1	TACC2	AF095791	Transforming, acidic coiled-coil containing protein 2	10q26	0.63
38264_at	RASGFR3	RABIF	U74324	RAB interacting factor	1q32-q41	0.63
32878_f_at	PPP1R3E	PPP1R3E	AA524802	Protein phosphatase 1, regulatory (inhibitor) subunit 3E	14q11.2	0.63
41646_at	JIK	TAOK3	AA576724	TAO kinase 3; STE20-like kinase	12q	0.63
39417_at	KIAA1028	CDC2L6	AB028951	Cell division cycle 2-like 6 (CDK8-like); Cyclin-dependent kinase (CDC2-like) 11	6q21	0.63
37159_at	DJ159A19.3	DJ159A19.3	U79259	Hypothetical protein DJ159A19.3	1p36.13	0.62
39013_at	ASP	APG5L	Y11588	APG5 autophagy 5-like (S. cerevisiae)	6q21	0.62
39780_at	CALNB	PPP3CB	M29551	Protein phosphatase 3 (formerly 2B), catalytic subunit, beta isoform (calcineurin A beta)	10q21-q22	0.62
39139_at	SPC18	SPC11L1	AI357653	Signal peptidase complex (18kD)	15q25.3	0.62
36184_at	PLOD	PLOD1	L06419	Procollagen-lysine 1, 2-oxoglutarate 5-dioxygenase 1; Lysyl hydroxylase	1p36.3-p36.2	0.62
40118_at	Zfp113	ZNF3	X07290	Zinc finger protein 3 (A8-51)	7q22.1	0.61
37045_at	KIAA0254	SNX19	D87443	Sorting nexin 19	11q25	0.60
32597_at	p130	RBL2	X76061	Retinoblastoma-like 2 (p130)	16q12.2	0.60
31892_at	hR-PTPu	PTPRM	X58288	Protein tyrosine phosphatase, receptor type, M	18p11.2	0.60
39346_at	p62	KHDRBS1	M88108	KH domain containing, RNA binding, signal transduction associated 1	1p32	0.60
34358_at	RPL23	MRPL23	Z49254	Mitochondrial ribosomal protein L23	11p15.5-p15.4	0.60
1825_at	SAR1	IQGAP1	L33075	IQ motif containing GTPase activating protein 1	15q26.1	0.59
32730_at	KIAA1750	TSPYL5	AL080059	TSPY-like 5	8q22.1	0.59
34218_at	SPEG	APEG1	U57099	Aortic preferentially expressed protein 1	2q35	0.59
41243_at	KIAA0447	SLC35E2	AB007916	Solute carrier family 35, member E2	1p36.33	0.59
35809_g_at			AL031681	Clone RP5-862K6 on chromosome 20q12-13	20q12-q13.1	0.59
34817_s_at	A2LP	ATXN2L	U70671	Ataxin 2-like	16p11	0.59
37659_at	p87/89	IMMT	L42572	Inner membrane protein, mitochondrial (mitofilin)	2	0.59
37646_at	KIAA0039	POLD3	D26018	Polymerase (DNA-directed), delta 3, accessory subunit	11q14	0.59
**39097_at**	**SON3**	**SON**	**X63753**	**SON DNA binding protein**	**21q22.11**	**0.59**
41708_at	KIAA1034	SATB2	AB028957	SATB family member 2	2q33	0.59
38142_at	ZNF-25	ZNF211	U38904	Zinc finger protein 211	19q13.4	0.58
39861_at	EMS1	CTTN	M98343	Cortactin	11q13	0.58
34676_at	KIAA1099	CENTG2	AB029022	Trinucleotide repeat containing 17	2p24.3-p24.1	0.57
35363_at	P72	DDX17	AL080113	DEAD (Asp-Glu-Ala-Asp) box polypeptide 17	22q13.1	0.57
35779_at	H1VPS45	VPS45A	AJ133421	Vacuolar protein sorting 45A (yeast)	1q21-q22	0.57
32628_at	DB1	ZNF161	D28118	Zinc finger protein 161	17q23.2	0.56
39718_r_at	RPL33L	MRPL33	N98607	Mitochondrial ribosomal protein L33	2p21	0.56
40801_at	DKFZP434C212	GAPVD1	AA643063	GTPase activating protein and VPS9 domains 1	9q33.3	0.56
35764_at	CX-ORF5 (71-7/	OFD1	Y15164	Oral-facial-digital syndrome 1	Xp22.2-p22.3	0.56
38733_at	Ku80	XRCC5	M30938	X-ray repair complementing defective repair in Chinese hamster cells 5 (double-strand-break rejoining; Ku autoantigen, 80kDa)	2q35	0.56
585_at	Ku80	XRCC5	M30938	X-ray repair complementing defective repair in Chinese hamster cells 5 (double-strand-break rejoining; Ku autoantigen, 80kDa)	2q35	0.56
39706_at	KIAA0636	CPNE3	AB014536	Copine III	8q21.3	0.55
36551_at	KIAA0373	Cep290	AL049382	Centrosome protein cep290	12q21.33	0.55
33296_at	KIAA0836	GLCE	AB020643	Glucuronyl C5-epimerase	15q23	0.55

Affy ID	Synonyms	Symbol	Genbank	Description	Chromosome	fold difference
37725_at	PPPIG	PPP1CC	X74008	Protein phosphatase 1, catalytic subunit, gamma isoform	12q24.1-q24.2	0.54
41860_at	LOC93081	LOC93081	AF070559	Hypothetical protein BC015148	13q33.1	0.54
41196_at	IMB1	KPNB1	L38951	Karyopherin (importin) beta 1	17q21.32	0.53
41205_at	EPVE6AP	UBE3A	U84404	Ubiquitin protein ligase E3A (human papilloma virus E6-associated protein, Angelman syndrome)	15q11-q13	0.53
34730_g_at	KIAA1114	TRO	AB029037	Trophinin	Xp11.22-p11.21	0.52
38837_at	KIAA1162	DJ971N18.2	W26226	Hypothetical protein LOC56255	20p12	0.52
192_at	TAFII55	TAF7	U18062	TAF7 RNA polymerase II, TATA box binding protein (TBP)-associated factor, 55kDa	5q31	0.52
33340_at	KIAA0438	PJA2	AB007898	Praja 2, RING-H2 motif containing	5q21.3	0.52
38365_at	ZWS1	PEX1	AF026086	Peroxisome biogenesis factor 1	7q21-q22	0.52
40994_at	GRK5	GRK5	L15388	G protein-coupled receptor kinase 5	10q24-qter	0.51
33907_at	eIF4G3	EIF4G3	AF012072	Eukaryotic translation initiation factor 4 gamma, 3	1p36.12	0.51
41669_at	KIAA0191	KIAA0191	D83776	Zinc finger, CCHC domain containing 11	1p32.3	0.51
33661_at	RPL5	RPL5	U66589	Similar to RIKEN cDNA 2900024C23	1p22.1	0.50
39692_at	DKFZP586F242	DKFZP586F	AL080209	CAMP responsive element binding protein 3-like 2	7q34	0.50
39136_at	ODD	OSR1	AB017642	Oxidative-stress responsive 1	2p24.1	0.50
32768_at	FLJ21007	TDRD3	AL048308	Tudor domain containing 3	13q21.2	0.50
33893_r_at	KAB	KIAA0470	AB007939	KARP-1-binding protein	1q44	0.49
35802_at	KIAA1014	FNBP4	AB023231	Formin binding protein 4	11p11.2	0.49
36532_at	INPP5H	SYNJ2	AF039945	Synaptojanin 2	6q25.3	0.49
34649_at	CSPG1	AGC1	M14219	Chondroitin/dermatan sulfate proteoglycan (PG40) core protein	15q26.1	0.49
35187_at			AL080216	DKFZp586K1123 cDNA	1	0.49
39983_at	FAP48	GLMN	U73704	Glomulin, FKBP associated protein	1p22.1	0.49
36692_at	MTMR9	MTMR9	AF052099	Myotubularin related protein 9	8p23-p22	0.47
1348_s_at	PCCA	PCCA	S79219	Propionyl Coenzyme A carboxylase, alpha polypeptide	13q32	0.47
1836_at	CYC1	CCNI	D50310	Cyclin I	4q21.1	0.46
34677_f_at	tl132	LOC220594	AJ012755	TL132 protein	17p11.2	0.46
39619_at	REEP2	LOC51308	AF070551	Chromosome 5 open reading frame 19	5q31	0.46
35142_at	DKFZP564D172	DKFZP564D	AF070617	Hypothetical protein FLJ12078	5q15	0.45
36161_at	AP2B1	AP2B1	M34175	Adaptor-related protein complex 2, beta 1 subunit	17q11.2-q12	0.44
40701_at	ISOT-3	USP13	U75362	Ubiquitin specific protease 13 (isopeptidase T-3)	3q26.2-q26.3	0.44
41747_s_at	MEF2A	MEF2A	U49020	Myocyte enhancer factor 2A	15q26	0.44
36225_s_at	SFPQ	SFPQ	W27611	Splicing factor proline/glutamine rich (polypyrimidine tract binding protein associated)	1p34.3	0.43
35625_at	CD97	CD97	X94630	CD97 transmembrane protein	19p13	0.43
34785_at	KIAA1025	THRAP2	AB028948	Thyroid hormone receptor associated protein 2	12q24.21	0.43
531_at	GliPR	GLIPR1	U16307	GLI pathogenesis-related 1 (glioma)	12q21.1	0.41
41872_at	DFNA5	DFNA5	AF073308	Deafness, autosomal dominant 5	7p15	0.37
36089_at	SAC2	VPS52	AB023183	Inositol polyphosphate-5-phosphatase F	6p21.3	0.36
32317_s_at	STP2	SULT1A2	U34804	Thermostable phenol sulfotransferase	16p12.1	0.34
33303_at	SSPN	SSPN	N21470	Sarcospan	12p11.2	0.29
33658_at	ZNF124	ZNF124	S54641	Zinc finger protein 124	1q44	0.29
41610_at	KIAA0533	LAMA5	AB011105	Laminin, alpha 5	20q13.2-q13.3	0.20
38170_at	clone 24583		AF070595	Clone 24583 mRNA sequence	9	0.18
37251_s_at	m6b1	GPM6B	AF016004	proteolipid 6B	Xp22.2	0.09
