# Supplementary material for: Cell type-specific over-expression of chromosome 21 genes in fibroblasts and fetal hearts with trisomy 21
Source: BMC Med Genet. 2006 Mar 15;7:24. doi: 10.1186/1471-2350-7-24 (PMC1435874; doi:10.1186/1471-2350-7-24)
Supplement: Additional File 3 — Genes over- or under-expressed in late passage compared to early passage fibroblasts, independently of +21 (2-way ANOVA, p < .001.). [file 1471-2350-7-24-S3.pdf]

Supplemental Table 3. Genes over- or under-expressed in late passage compared to early passage fibroblasts, independently of +21 (2-way ANOVA, p<.001.)

Affy ID	2-way ANOVA p value; late vs early passage independent of +21	fold difference late vs early passage C	fold difference late vs early passage +21	Symbol	Synonym	Genbank	Description
41814_at	0.00056	4.12	2.37	FUCA1	FUCA1	M29877	Fucosidase, alpha-L- 1, tissue
36136_at	0.00026	3.87	3.38	PIG11	PIG11	AF010315	Tumor protein p53 inducible protein 11
34842_at	0.00029	3.76	2.69	SNRPN	SNRPN	U41303	SNRPN upstream reading frame
1120_at	0.00021	3.12	2.16	GSTM3	GSTM3	J05459	Glutathione S-transferase M3 (brain)
35576_f_at	0.00009	3.09	3.11	H2BFC	H2BFC	AL009179	H2B histone family, member C
41237_at	0.00032	3.03	1.85	HLA-A	HLA-A	D32129	Major histocompatibility complex, class I, A
32822_at	0.00064	3.01	1.41	ANT1	SLC25A4	J02966	mitochondrial carrier; adenine nucleotide translocator
40120_at	0.00009	2.99	2.40	HAGH1	HAGH	X90999	Hydroxyacylglutathione hydrolase
36347_f_at	0.00003	2.81	4.12	HIST1H2BN	H2BFD	AA873858	Histone 1, H2bn
1243_at	0.00008	2.69	2.74	DDB2	DDB2	U18300	Damage-specific DNA binding protein 2, XP group E gene
37022_at	0.00014	2.69	3.81	PRELP	PRELP	U41344	leucine-rich repeat protein of articular cartilage; prolargin
39340_at	0.00008	2.63	2.37	HEXA	HEXA	M16424	beta-hexosaminidase alpha chain
35329_at	0.00002	2.58	2.42	CYB5R1	CYB5R1	AF091084	NAD(P)H:quinone oxidoreductase type 3, polypeptide A2
39369_at	0.00014	2.57	2.11	KIAA0935	KIAA0935	AB023152	Mannosidase, alpha, class 2B, member 2
40454_at	0.00084	2.47	1.65	hFat	FAT	X87241	FAT tumor suppressor homolog 1 (Drosophila)
41627_at	0.00045	2.38	1.97	SDF2	SDF2	D50645	Stromal cell-derived factor 2
32566_at	0.00044	2.36	1.64	FLJ22678	FLJ22678	AA165701	Chondroitin polymerizing factor
32819_at	0.00001	2.32	2.79	HIRIP1	H2BFT	AJ223352	Histone 1, H2bk
31522_f_at	0.00011	2.31	2.82	H2B/g	H2BFG	Z80779	H.sapiens H2B/g gene.
37351_at	0.00098	2.30	1.63	UP	UP	X90858	uridine phosphorylase
37466_at	0.00095	2.26	1.94	RAB7L1	RAB7L1	D84488	RAB7, member RAS oncogene family-like 1
1119_at	0.00010	2.26	1.59	RPA2	RPA2	J05249	Replication protein A2, 32kDa
33780_at	0.00098	2.24	4.03	SYB1	VAMP1	M36200	synaptobrevin 1
40136_at	0.00089	2.24	1.71	KIAA0676	KIAA0676	AB014576	KIAA0676 protein
41724_at	0.00034	2.23	1.59	BAP31	BCAP31	X81817	BAP31
38274_at	0.00064	2.22	1.60	PL6	PL6	U09584	Placental protein 6
41060_at	0.00035	2.21	1.67	CCNE1	CCNE1	M74093	Cyclin E1
32610_at	0.00095	2.21	1.47	ril	RIL	X93510	PDZ and LIM domain 4
38439_at	0.00042	2.18	2.03	NFE2L1	NRF1	L24123	NRF1
40427_at	0.00057	2.18	1.64	COX17	COX17	AA149486	COX17
34780_at	0.00096	2.17	1.83	KIAA0315	PLXNB2	AB002313	Plexin B2
32094_at	0.00092	2.14	1.64	CHST3	CHST3	AB017915	Carbohydrate (chondroitin 6) sulfotransferase 3
34285_at	0.00007	2.12	1.87	KIAA0795	KIAA0795	AB018338	Kelch-like 18 (Drosophila)
38188_s_at	0.00065	2.11	1.95	MAN2A2	MAN2A2	L28821	Mannosidase, alpha, class 2A, member 2
36989_at	0.00015	2.07	1.46	DAG1	DAG1	L19711	Dystroglycan 1 (dystrophin-associated glycoprotein 1)
37308_at	0.00002	2.06	1.41	GPR107	GPR107	AI888084	LOC441469
33860_at	0.00068	2.06	1.44	KIAA0462	RBAF600	AB007931	Retinoblastoma-associated factor 600
38625_g_at	0.00048	2.04	2.50	KCC1	SLC12A4	AF054506	Solute carrier family 12 (potassium/chloride transporters), member 4
35821_at	0.00099	1.99	1.51	HDAC3	HDAC3	U75697	Histone deacetylase 3
35771_at	0.00013	1.98	1.58	DEAF1	DEAF1	AF049460	Deformed epidermal autoregulatory factor 1 (Drosophila)
36194_at	0.00037	1.98	2.07	A2M	LRPAP1	M63959	Low density lipoprotein receptor-related protein associated protein 1
37045_at	0.00068	1.96	1.14	KIAA0254	KIAA0254	D87443	Similar to Sorting nexin 19
32095_at	0.00096	1.96	1.99	KIAA0724	IMP13	AB018267	Importin 13
111_at	0.00072	1.96	1.44	RABGGTA	RABGGTA	Y08200	Rab geranylgeranyltransferase, alpha subunit
37395_at	0.00003	1.95	1.86	ATP6V1F	ATP6V1F	D49400	ATPase, H+ transporting, lysosomal 14kDa, V1 subunit F
39693_at	0.00003	1.95	2.26	MGC5508	MGC5508	N53547	Hypothetical protein MGC5508
34892_at	0.00031	1.95	1.50	TNFRSF10B	TNFRSF10B	AF016266	Tumor necrosis factor receptor superfamily, member 10b
32321_at	0.00066	1.95	1.97	HLA-E	HLA-E	X56841	Major histocompatibility complex, class I, E
32799_at	0.00000	1.94	1.85	SCAMP3	SCAMP3	AF023268	
40815_g_at	0.00070	1.92	1.63	IDS	IDS	L40586	Iduronate 2-sulfatase (Hunter syndrome)
41417_at	0.00014	1.92	1.44	MIR16	MIR16	AC003108	
38670_at	0.00023	1.90	2.23	ZNF313	ZNF313	AL031685	
37320_at	0.00092	1.90	1.24	KIAA0024	PTDSS1	D14694	Phosphatidylserine synthase 1
39783_at	0.00063	1.90	1.56	KIAA0100	KIAA0100	D43947	KIAA0100 gene product
1850_at	0.00069	1.89	1.26	hmlh1	MLH1	U07418	MutL homolog 1, colon cancer, nonpolyposis type 2
38373_g_at	0.00099	1.89	1.44	LOC91966	IDS	U66042	Chromosome X open reading frame 40
35770_at	0.00006	1.88	1.69	ATP6IP1	VATPS1	D16469	Human mRNA for ORF, Xq terminal portion.
34868_at	0.00087	1.88	2.38	KIAA1089	KIAA1089	AB029012	Est1p-like protein B
35365_at	0.00081	1.87	1.68	ILK	ILK	U40282	Integrin-linked kinase
38372_at	0.00014	1.85	1.21	LOC91966	IDS	U66042	Chromosome X open reading frame 40
631_g_at	0.00001	1.85	1.39	DCTD	DCTD	L39874	Homo sapiens deoxycytidylate deaminase gene, complete cds.
38421_at	0.00016	1.85	2.00	DKFZp451J0118	DKFZp451J0118	AF070546	Taxilin
36795_at	0.00005	1.83	1.92	GLBA	PSAP	J03077	Prosaposin (variant Gaucher disease and variant metachromatic leukodystrophy)
39339_at	0.00022	1.83	1.47	KIAA0792	KIAA0792	AB018335	KIAA0792 gene product
2056_at	0.00056	1.83	1.57	FGFR1	FGFR1	M34641	Fibroblast growth factor receptor 1 (fms-related tyrosine kinase 2, Pfeiffer syndrome)
38055_at	0.00027	1.82	1.69	POLR2J	DKFZp434K1815	AI683748	Polymerase (RNA) II (DNA directed) polypeptide J, 13.3kDa
39130_at	0.00081	1.82	1.86	KIAA0770	VPS39	AB018313	Vacuolar protein sorting 39 (yeast)
34651_at	0.00009	1.82	1.94	COMT	COMT	M58525	Catechol-O-methyltransferase
31524_f_at	0.00020	1.82	2.75	H2B/k	H2BFK	Z80782	H.sapiens H2B/k gene.
38697_at	0.00005	1.81	1.41	DKFZp566C243	DKFZP566C243	AL050274	Chromosome 6 open reading frame 109
35617_at	0.00034	1.81	1.63	MAPK7	MAPK7	U29725	Mitogen-activated protein kinase 7
37274_at	0.00004	1.80	1.39	Btd	BTD	AF018631	biotin-amide amidohydrolase; biotinidase
31523_f_at	0.00025	1.80	2.44	H2B/h	H2BFH	Z80780	H.sapiens H2B/h gene.
34861_at	0.00052	1.77	1.40	GOLGA3	GOLGA3	D63997	Golgi autoantigen, golgin subfamily a, 3
317_at	0.00039	1.76	1.45	LGMN	LGMN	D55696	Legumain
635_s_at	0.00014	1.75	2.27	PP2A	PPP2R5B	L42374	Protein phosphatase 2, regulatory subunit B (B56), beta isoform
33361_at	0.00081	1.75	1.88	BSCL2	BSCL2	AF052149	Bernardinelli-Seip congenital lipodystrophy 2 (seipin)
38394_at	0.00060	1.74	1.36	KIAA0089	KIAA0089	D42047	Glycerol-3-phosphate dehydrogenase 1-like
1911_s_at	0.00013	1.73	1.72	gadd45	GADD45A	M60974	Growth arrest and DNA-damage-inducible, alpha
36154_at	0.00085	1.72	1.52	KIAA0263	IHPK1	D87452	Inositol hexaphosphate kinase 1
35176_at	0.00013	1.71	1.51	DPAGT1	DPAGT1	Z82022	H.sapiens mRNA for GlcNac-1-P transferase.
35749_at	0.00048	1.71	1.65	TADA3L	TADA3L	AF069733	Transcriptional adaptor 3 (NGG1 homolog, yeast)-like
31673_s_at	0.00002	1.71	1.32	cell adhesion regu	SPG7	X65784	H.sapiens CAR gene.
33749_at	0.00004	1.70	2.18	P53TG1	TP53TG1	AB007455	TP53 activated protein 1
37666_at	0.00066	1.69	1.38	PSMB5	PSMB5	D29011	Human mRNA for proteasome subunit X, complete cds.
32856_at	0.00024	1.69	1.34	KIAA0819	KIAA0819	AB020626	KIAA0819 protein
33875_at	0.00006	1.69	2.02	ATP6V0E	ATP6V0E	AI547262	ATPase, H+ transporting, lysosomal 9kDa, V0 subunit e
40546_s_at	0.00070	1.68	1.50	NDUFA2	NDUFA2	AF047185	NADH-ubiquinone oxidoreductase subunit CI-B8
100_g_at	0.00086	1.65	1.89	RABGGTA	RABGGTA	Y08200	Rab geranylgeranyltransferase, alpha subunit
35347_at	0.00000	1.65	1.80	UPH1	EFEMP2	AF093119	EGF-containing fibulin-like extracellular matrix protein 2

Affy ID	2-way ANOVA p value; late vs early passage independent of +21	fold difference late vs early passage C	fold difference late vs early passage +21	Symbol	Synonym	Genbank	Description
37098_at	0.00021	1.65	1.83	PPOX	PPOX	D38537	Protoporphyrinogen oxidase
39517_at	0.00041	1.64	1.37	HTGN29	HTGN29	AF035313	Chromosome 5 open reading frame 15
39089_at	0.00008	1.64	1.83	nm23-H4	NME4	Y07604	Non-metastatic cells 4, protein expressed in
36473_at	0.00033	1.63	1.54	KIAA1003	USP20	AB023220	Ubiquitin specific protease 20
35840_at	0.00007	1.62	1.83	DKFZp566H073	DKFZP566H073	AL050060	Ring finger protein 167
35767_at	0.00002	1.59	1.55	GABARAPL2	GABARAPL2	AI565760	GABA(A) receptor-associated protein-like 2
32716_at	0.00021	1.58	1.45	DGKA	DGKA	X62535	Diacylglycerol kinase, alpha 80kDa
36996_at	0.00099	1.58	1.52	OS-9	OS-9	U41635	Amplified in osteosarcoma
39341_at	0.00049	1.56	1.56	TRIP6	TRIP6	AJ001902	Thyroid hormone receptor interactor 6
36603_at	0.00024	1.55	1.23	KIAA0219	GCN1L1	D86973	GCN1 general control of amino-acid synthesis 1-like 1
37998_at	0.00024	1.55	1.34	HLP	SKIV2L	U09877	Superkiller viralicidic activity 2-like (S. cerevisiae)
39729_at	0.00010	1.55	1.44	NKEFB	PRDX2	L19185	Human natural killer cell enhancing factor (NKEFB)
39795_at	0.00073	1.55	1.42	KIAA0109	AP2M1	D63475	Adaptor-related protein complex 2, mu 1 subunit
1885_at	0.00098	1.55	1.50	ERCC3	ERCC3	M31899	Excision repair cross-complementing, complementation group 3
39383_at	0.00009	1.55	1.59	KIAA0422	ADCY6	AB007882	Adenylate cyclase 6
40465_at	0.00045	1.54	1.47	U5-100K	U5-100K	AF026402	DEAD (Asp-Glu-Ala-Asp) box polypeptide 23
39521_at	0.00086	1.54	2.17	hKCC1	SLC12A4	U55054	Solute carrier family 12 (potassium/chloride transporters), member 4
38067_at	0.00079	1.53	1.26	KIAA0202	38968	D86957	Septin 8
41757_at	0.00028	1.53	1.88	VAPB	VAPB	W25933	Transcribed locus
38517_at	0.00049	1.53	1.92	ISGF3-gamma	ISGF3G	M87503	Interferon-stimulated transcription factor 3, gamma 48kDa
34409_at	0.00009	1.52	1.70	DKFZp564C1940	LRP10	AL080164	Low density lipoprotein receptor-related protein 10
1706_at	0.00089	1.52	1.49	A-RAF-1	ARAF1	U01337	Human Ser/Thr protein kinase (A-RAF-1) gene
39348_at	0.00096	1.51	1.39	HRMT1L1	HRMT1L1	X99209	HMT1 hnRNP methyltransferase-like 1 (S. cerevisiae)
40370_f_at	0.00068	1.50	2.13	HLA-G	HLA-G	M90683	HLA-G histocompatibility antigen, class I, G
40369_f_at	0.00006	1.50	2.01	HLA-G	HLA-G	AL022723	
38425_at	0.00047	1.50	1.53	HMGCL	HMGCL	U49719	HMG CoA lyase; Human hydroxymethylglutaryl-CoA lyase
37362_at	0.00040	1.49	1.25	RAB5B	RAB5B	X54871	RAB5B, member RAS oncogene family
40498_g_at	0.00034	1.49	1.48	NPR2L	NPR2L	AF040707	Tumor suppressor candidate 4
34413_at	0.00045	1.48	1.33	NUDEL	NUDEL	AF038203	NudE nuclear distribution gene E homolog like 1 (A. nidulans)
890_at	0.00050	1.47	1.23	UBE2A	UBE2A	M74524	Ubiquitin-conjugating enzyme E2A (RAD6 homolog)
34679_at	0.00053	1.46	1.49	bcr	BCR	X02596	Breakpoint cluster region
36955_at	0.00054	1.46	1.48	C5orf8	C5orf8	U10362	Lectin, mannose-binding 2
40462_at	0.00032	1.44	1.54	C20orf188	C20orf188	AF055022	Transient receptor potential cation channel, subfamily C, member 4 associated protein
31605_at	0.00003	1.44	1.43	LOC171220	LOC171220	U72518	RPL13-2 pseudogene
31839_at	0.00084	1.44	1.67	SF4; RBP; F2385	SF4	AC004475	Similar to novel proteins encoded by (AC004447) KIAA0365
38725_s_at	0.00026	1.43	1.37	DPM2	DPM2	N36295	Dolichyl-phosphate mannosyltransferase polypeptide 2, regulatory subunit
36583_at	0.00095	1.43	2.02	SNX1	SNX1	U53225	Human sorting nexin 1 (SNX1) mRNA, complete cds.
32758_g_at	0.00079	1.42	1.45	RAE1	RAE1	U84720	RAE1 RNA export 1 homolog (S. pombe)
32980_f_at	0.00001	1.41	2.25	HIST1H2BC	H2BFL	AI688098	Histone 1, H2bc
39811_at	0.00084	1.41	1.28	MGC2749	MGC2749	AA402538	Hypothetical protein MGC2749
38412_at	0.00066	1.40	1.22	HCG V	PPP1R11	U53588	Homo sapiens MHC class 1 region.
37240_at	0.00052	1.38	1.30	HO3	HARSL	U18937	Histidyl-tRNA synthetase-like
40360_at	0.00080	1.38	1.27	P3; DXS253E	P3	X12458	unnamed protein product; P3 protein (AA 1-1382); Human P3 gene.
752_s_at	0.00030	1.25	1.62	HSPF1	DNAJB1	D85429	Hsp40;Similar to bacterial DnaJ heat shock protein
37922_at	0.00081	1.21	1.64	TCN2	TCN2	L02648	Transcobalamin II; macrocytic anemia
41773_at	0.00072	1.21	1.58	PCOLN3	PCOLN3	U58048	Procollagen (type III) N-endopeptidase
1707_g_at	0.00074	1.15	2.31	A-RAF-1	ARAF1	U01337	Human Ser/Thr protein kinase (A-RAF-1) gene
1348_s_at	0.00078	0.79	0.42	metastasis-associ	PCCA	S79219	Propionyl Coenzyme A carboxylase, alpha polypeptide
35293_at	0.00045	0.79	0.13	SSA2	SSA2	J04137	Sjogren syndrome antigen A2 (60kDa, ribonucleoprotein autoantigen SS-A/Ro)
37162_at	0.00082	0.71	0.62	H4(D10S170)	D10S170	S72869	Coiled-coil domain containing 6
31463_s_at	0.00002	0.67	0.62			AL022097	Human DNA sequence from clone RP1-256G22 on chromosome 6p24.1-25.3
38150_at	0.00071	0.62	0.42	MTAP	MTAP	U22233	Methylthioadenosine phosphorylase
31675_s_at	0.00015	0.52	0.48	PTH2	PTENP1	AF019083	Phosphatase and tensin homolog, pseudogene 1
1952_s_at	0.00071	0.47	0.44	Smad5	MADH5	AF010607	SMAD5
40990_at	0.00065	0.33	0.46	TM4SF9	TM4SF9	AF065389	Transmembrane 4 superfamily member 9
33817_at	0.00089	0.32	0.30	D10S102		S63912	Heterogeneous nuclear ribonucleoprotein A3 pseudogene 1
34279_at	0.00058	0.30	0.45	MGC8902	FLJ20719	AL050141	AG1
