# Supplementary material for: Cell type-specific over-expression of chromosome 21 genes in fibroblasts and fetal hearts with trisomy 21
Source: BMC Med Genet. 2006 Mar 15;7:24. doi: 10.1186/1471-2350-7-24 (PMC1435874; doi:10.1186/1471-2350-7-24)
Supplement: Additional File 4 — Genes over- or under-expressed in fetal hearts with +21. ANOVA, p < .05. [file 1471-2350-7-24-S4.pdf]

Supplemental Table 4. Genes over- or under-expressed in fetal hearts with +21. ANOVA, p<.05.

Affy ID	Synonyms	Symbol	Genbank	Description	Chromosome	fold difference
33455_at	ALDOB	ALDOB	X02747	Aldolase B, fructose-bisphosphate	9q21.3-q22.2	4.56
32027_at	PDZK1	PDZK1	AF012281	PDZ domain containing 1	1q21	3.99
40954_at	FXYD2	FXYD2	H94881	FXYD domain containing ion transport regulator 2	11q23	3.65
39151_at	PLAC3; PAPPE	PAPPA2	AL031290	Clone RP4-774I24 on chromosome 1q24.1-24.3	1q23-q25	3.47
425_at	ISG12	IFI27	X67325	Interferon, alpha-inducible protein 27	14q32	3.47
**38384_at**	**GART**	**GART**	**X54199**	**Phosphoribosylglycinamide formyltransferase, phosphoribosylglycinamide synthetase, phosphoribosylaminoimidazole synthetase**	**21q22.11**	**3.02**
291_s_at	M1S1	TACSTD2	J04152	GA733-1 protein precursor; Human gastrointestinal tumor-associated antigen GA733-1 protein gene, complete cds, clone 05516.	1p32-p31	2.75
35692_at	DKFZp586E162	RIS1	AL080235	Ras-induced senescence 1	3p21.3	2.63
38469_at	TM4SF3	TSPAN8	M35252	Transmembrane 4 superfamily member 3	12q14.1-q21.1	2.60
40201_at	ddc	DDC	M76180	Dopa decarboxylase (aromatic L-amino acid decarboxylase)	7p11	2.54
39660_at	DEFB1	DEFB1	AI309115	Defensin, beta 1	8p23.2-p23.1	2.34
40071_at	CYP1B1	CYP1B1	U03688	Cytochrome P450, family 1, subfamily B, polypeptide 1	2p21	2.33
31737_at	ACTA1	ACTA1	J00068	Actin, alpha 1, skeletal muscle	1q42.13-q42.2	2.29
828_at	PTGER2	PTGER2	U19487	Prostaglandin E receptor 2 (subtype EP2), 53kDa	14q22	2.24
37202_at	F2	F2	J00307	Prothrombin	11p11-q12	2.15
859_at	CYP1B1	CYP1B1	U03688	Cytochrome P450, family 1, subfamily B, polypeptide 1	2p21	2.15
36256_at	LSAMP	LSAMP	U41901	Limbic system-associated membrane protein	3q13.2-q21	2.10
**37827_r_at**	**C21orf5**	**C21orf5**	**AJ237839**	**Chromosome 21 open reading frame 5, transcription factor?**	**21q22.2**	**2.09**
36245_at	HTR2B	HTR2B	X77307	5-hydroxytryptamine (serotonin) receptor 2B	2q36.3-q37.1	2.05
33364_at	PDE4DIP	PDE4DIP	U51694	Phosphodiesterase 4D interacting protein	1q12	2.03
39260_at	MCT	SLC16A4	U59185	Solute carrier family 16 (monocarboxylic acid transporters) 4	1p13.3	2.00
35680_r_at	DPP6	DPP6	M96860	Dipeptidylpeptidase 6	7q36.2	1.99
34316_at	RPS15A	RPS15A	W52024	Ribosomal protein S15a	16p	1.96
37871_at	IAPP; DAP; AM	IAPP	X68830	Islet amyloid polypeptide	12p12.3-p12.1	1.94
37459_at	COL8A1	COL8A1	X57527	Collagen, type VIII, alpha 1	3q12.3	1.91
38201_at	ECA39	BCAT1	U21551	Branched chain aminotransferase 1, cytosolic	12pter-q12	1.88
40413_at	MRPS29	DAP3	U18321	Death associated protein 3	1q21-q22	1.88
33203_s_at	FREAC-4	FOXD1	U59831	Forkhead related activator 4 (FREAC-4)	5q12-q13	1.87
**34559_at**		**LOC388818**	**H55746**	**Similar to RIKEN cDNA 2310002B14**	**21q22.11**	**1.85**
38430_at	FABP4	FABP4	AA128249	Fatty acid binding protein 4, adipocyte	8q21	1.84
39248_at	AQP3	AQP3	N74607	Aquaporin 3	9p13	1.82
39999_at	Mch4	CASP10	U60519	Caspase 10	2q33-q34	1.82
37399_at	KIAA0119	AKR1C3	D17793	Aldo-keto reductase family (3-alpha hydroxysteroid dehydrogenase, type II)	10p15-p14	1.81
32486_at	CKMM	CKM	AC005781	Creatine kinase, muscle; M chain	19q13.2-q13.3	1.81
35422_at	MAP2	MAP2	U01828	Microtubule-associated protein 2	2q34-q35	1.78
32818_at	TNC	TNC	X78565	Tenascin C (hexabrachion	9q33	1.77
38978_at	PAIP1	PAIP1	AF013758	Poly(A) binding protein interacting protein	5p12	1.75
**31477_at**	**TFF3**	**TFF3**	**L08044**	**Trefoil factor 3 (intestinal)**	**21q22.3**	**1.75**
116_at	PRKAR2A	PRKAR2A	X14968	Protein kinase, cAMP-dependent, regulatory, type II, alpha	3p21.3-p21.2	1.73
37687_i_at	CD32	FCGR2A	M31932	Fc fragment of IgG, low affinity IIa, recepto	1q23	1.73
1574_s_at	IL4	IL4	M13982	Interleukin 4	5q31.1	1.72
37284_at	CD100	SEMA4D	U60800	Semaphorin 4D	9q22-q31	1.72
39674_r_at	ECM2	ECM2	AB011792	Extracellular matrix protein 2	9q22.3	1.72
1008_f_at	Pkr	EIF2AK2	U50648	Interferon-inducible RNA-dependent protein kinas	2p22-p21	1.71
39968_at	LTC4S	LTC4S	U50136	Leukotriene C4 synthase	5q35	1.70
245_at	LNHR	SELL	M25280	Selectin L (lymphocyte adhesion molecule 1	1q23-q25	1.70
33462_at	GPR105	P2RY14	D13626	Purinergic receptor P2Y, G-protein coupled, 14	3q21-q25	1.66
38919_at	KIAA1009	C6orf84	AB023226	Chromosome 6 open reading frame 84	6q14.3	1.66
**38738_at**	**SMT3A**	**SUMO3**	**X99584**	**SMT3 suppressor of mif two homolog 3**	**21q22.3**	**1.66**
36155_at	Testican-2	KIAA0275	D87465	Sparc/osteonectin, cwcv and kazal-like domains proteoglycan (testican) 2	10pter-q25.3	1.63
1300_at	DKFZp781P091	XRCC2	Y08837	X-ray repair complementing defective repair in Chinese hamster cells 2		1.62
**306_s_at**	**HMG14**	**HMGN1**	**J02621**	**High-mobility group nucleosome binding domain 1**	**21q22.2**	**1.62**
31623_f_at	MT1	MT1A	K01383	Metallothionein-I-A gene	16q13	1.61
37835_at	CD1, a1 domain	CD1C	M28827	CD1C antigen, c polypeptide	1q22-q23	1.61
32250_at	CFH	HF1	X07523	Complement factor H	1q32	1.61
40587_s_at	P18	EEF1E1	AF054186	Eukaryotic translation elongation factor 1 epsilon	6p24.3-p25.1	1.60
1975_s_at	IGF-I	IGF1	X03563	Insulin-like growth factor	12q22-q23	1.60
38338_at	RRAS	RRAS	AI201108	Related RAS viral (r-ras) oncogene homolog	19q13.3-qter	1.58
36839_at	HsCDC6	CDC6	U77949	CDC6 cell division cycle 6 homolog (S. cerevisiae	17q21.3	1.57
**36107_at**	**F6**	**ATP5J**	**AA845575**	**ATP synthase, H+ transporting, mitochondrial F0 complex, subunit F6**	**21q21.1**	**1.55**
201_s_at	B2M	B2M	S82297	Beta-2-microglobulin	15q21-q22.2	1.54
33267_at	ATPASEII	ATP8A1	AF035315	ATPase, aminophospholipid transporter (APLT), Class I, type 8A, member 1	4p14-p12	1.54
**39767_at**	**KIAA0002**	**CCT8**	**D13627**	**Chaperonin containing TCP1, subunit 8 (theta**	**21q22.11**	**1.53**
121_at	PAX8	PAX8	X69699	Paired box gene 8	2q12-q14	1.53
34708_at	HAKA1	FCN3	D88587	Ficolin/Opsonin p35 lectin family; Hakata antige	1p36.11	1.52
37177_at	LFA-3	CD58	Y00636	CD58 antigen, (lymphocyte function-associated antigen 3)	1p13	1.52
33358_at	KIAA1157	PPM1H	W29087	Protein phosphatase 1H (PP2C domain containing	12q14.1-q14.2	1.52
755_at	Insp3r1	ITPR1	D26070	Inositol 1,4,5-triphosphate receptor, type 1	3p26-p25	1.52
**39348_at**	**HRMT1L1**	**HRMT1L1**	**X99209**	**HMT1 hnRNP methyltransferase-like 1**	**21q22.3**	**1.52**
38700_at	CSRP	CSRP1	M33146	Cysteine and glycine-rich protein 1	1q32	1.51
33327_at	KIAA0954	C11orf9	AB023171	Chromosome 11 open reading frame 9	11q12-q13.1	1.51
**36039_s_at**	**21-garp**	**SH3BGR**	**X93498**	**SH3 domain binding glutamic acid-rich protein**	**21q22.3**	**1.51**
1440_s_at	Fas/Apo-1	TNFRSF6	X83490	Tumor necrosis factor receptor 6	1p33	1.51
**37218_at**	**TOFA**	**BTG3**	**D64110**	**BTG family, member 3**	**21q21.1-q21.2**	**1.49**
41244_f_at	PP-1B	PPP1CB	X80910	Protein phosphatase 1, catalytic subunit, beta isoform	2p23	1.49
38154_at			AF038199	Similar to hypothetical protein LOC283585		1.48
35229_at	CPT1	CPT1A	L39211	Carnitine palmitoyltransferase 1A (liver	11q13.1-q13.2	1.48
33179_at	IPP2	PPP1R2	AI625801	Protein phosphatase 1, regulatory (inhibitor) subunit 2	3q29	1.47
39594_f_at	MT1H	MT1H	R93527	Metallothionein 1H	16q13	1.47
40858_at	PSBG1	PSG1	M34715	Pregnancy specific beta-1-glycoprotein 1	19q13.2	1.47
40074_at	NMDMC	MTHFD2	X16396	Methylene tetrahydrofolate dehydrogenase (NAD+ dependent); methenyltetrahydrofolate cyclohydrolase	2p13.1	1.46
34995_at	CRLR	CALCRL	L76380	Calcitonin receptor-like	2q32.1	1.46

Affy ID	Synonyms	Symbol	Genbank	Description	Chromosome	fold difference
37756_at	RYK1	RYK	S59184	RYK receptor-like tyrosine kinase	3q22	1.45
**35816_at**	**cystatin B**	**CSTB**	**U46692**	**EPM1 disease gene; cysteine protease inhibitor; Cystatin B**	**21q22.3**	**1.44**
36204_at	LAR	PTPRF	Y00815	Protein tyrosine phosphatase, receptor type, I	1p34	1.44
37231_at	KIAA0008	DLG7	D13633	Discs, large homolog 7 (Drosophila	14q22.3	1.44
**39005_s_at**	**KIAA0714**	**ZNF294**	**AB018257**	**Zinc finger protein 294**	**21q22.11**	**1.43**
36042_at	trkB	NTRK2	X75958	Neurotrophic tyrosine kinase, receptor, type [illegible]	9q22.1	1.42
**33228_g_at**	**IL-10R2**	**IL10RB**	**AI984234**	**Interleukin 10 receptor, beta**	**21q22.11**	**1.41**
**879_at**	**MX2**	**MX2**	**M30818**	**Myxovirus (influenza virus) resistance [illegible]**	**21q22.3**	**1.40**
38066_at	DLA4	NQO1	M81600	NAD(P)H:quinone oxireductase	16q22.1	1.40
34667_at	NFX2; MGC203	NFX1	U15306	Nuclear transcription factor, X-box binding	9p13.3	1.40
32112_s_at	ST4	AIM1	AI800499	Absent in melanoma 1	6q21	1.39
38164_at	COD1	RPGR	U57629	Retinitis pigmentosa GTPase regulato	Xp11.4	1.39
37902_at	CRYZ	CRYZ	L13278	Crystallin, zeta (quinone reductase	1p31-p22	1.39
36332_at	SNAT; AA-NAT	AANAT	U40391	Serotonin N-acetyltransferase	17q25	1.39
39917_at	GCP2	TUBGCP2	AI961040	Tubulin, gamma complex associated protein [illegible]	10q26.3	1.38
**32168_s_at**	**DSCR1**	**DSCR1**	**U85267**	**Down syndrome critical region gene 1**	**21q22.12**	**1.37**
37716_at	MOX2	CD200	X05323	MOX2 gene; OX-2 membrane glycoprotein	3q12-q13	1.36
37264_at	pHZ-10	ZNF131	U09410	Zinc finger protein 131 (clone pHZ-10	5p12-p11	1.35
35537_at	DCR2	TNFRSF10D	AF029761	Tumor necrosis factor receptor superfamily, member 10d, decoy with truncated death domain	8p21	1.35
34160_at	beta-actin	ACTG1	X04098	Actin, gamma 1	17q25	1.34
32272_at	K-ALPHA-1	K-ALPHA-1	K00558	Tubulin, alpha, ubiquitous	12q13.12	1.34
36181_at	MLN 50; Lasp-1	LASP1	X82456	LIM and SH3 protein 1	17q11-q21.3	1.33
38826_at	KIAA0128	SEPT6	D50918	Septin 6	Xq24	1.33
**32236_at**	**UBC7**	**UBE2G2**	**AF032456**	**Ubiquitin conjugating enzyme G2**	**21q22.3**	**1.32**
31622_f_at	MT1F	MT1F	M10943	Metallothionein IF	16q13	1.31
36160_s_at	PTPRP	PTPRN2	U81561	Protein tyrosine phosphatase, receptor type, N polypeptide 2	7q36	1.31
32443_at	HZF22	ZNF157	U28687	Zinc finger protein 157 (HZF22	Xp11.2	1.31
457_s_at	UBL1	SUMO-1	U67122	SMT3 suppressor of mif two 3 homolog 1 (yeast	2q33	1.31
185_at	Nova-1	NOVA1	U04840	Neuro-oncological ventral antigen [illegible]	14q	0.75
41715_at	C2-PI3K	PIK3C2B	Y11312	Phosphoinositide-3-kinase, class 2, beta polypeptide	1q32	0.75
36909_at	Wee1 Hu	WEE1	X62048	WEE1 homolog (S. pombe)	11p15.3-p15.1	0.75
35289_at	GAPCENA	RABGAP1	AJ011679	RAB GTPase activating protein 1	9q33.2-q33.3	0.75
35638_at	CBFA2T1	RUNX1T1	D43638	Core-binding factor, runt domain, alpha subunit 2; translocated to, 1 (cyclin D-related)	8q22	0.74
35408_i_at	KOX7	ZNF44	X16281	Hypothetical protein DKFZp686L21136	19p13.2	0.74
37561_at			AL031778	Clone RP1-34B21 on chromosome 6p12.1-21.1	6p12.1-p21.1	0.74
39058_at	MDB	ABR	U01147	Active BCR-related gene	17p13.3	0.74
38036_at	RPI	RPIA	L35035	Ribose 5-phosphate isomerase A (ribose 5-phosphate epimerase)	2p11.2	0.74
40905_s_at	DKFZp566J153	PRPF31	AL050369	PRP31 pre-mRNA processing factor 31 homolog (yeast)	19q13.42	0.74
40102_at	KIAA0772	OSBPL2	AB018315	Oxysterol binding protein-like [illegible]	20q13.3	0.74
36115_at	clk3	CLK3	L29217	CDC-like kinase 3	15q24	0.74
33333_at	KIAA0403	PIP3-E	AB007863	Phosphoinositide-binding protein PIP3-E	6q25.2	0.74
39794_at	KIAA0055	USP8	D29956	Ubiquitin specific protease 8	15q21.2	0.74
37410_at	MED24	SURF5	AJ224358	Surfeit 5	9q34.2	0.73
1274_s_at	E2-CDC34	CDC34	L22005	Cell division cycle 34	19p13.3	0.73
40801_at	DKFZP434C212	GAPVD1	AA643063	DKFZP434C212 protein	9q33.3	0.73
33457_at	KIAA1105	RAP140	AB029028	Retinoblastoma-associated protein 140	3p14.3	0.73
33881_at	ACSL3	FACL3	AA977580	Acyl-CoA synthetase long-chain family member [illegible]	2q34-q35	0.73
1797_at	INK4D	CDKN2D	U40343	Cyclin-dependent kinase inhibitor 2D (p19, inhibits CDK4)	19p13	0.73
34169_s_at	OCRL1	OCRL	U57627	Oculocerebrorenal syndrome of Lowe	Xq25-q26.1	0.73
39777_at	KIAA0916	MYCBP2	AF075587	MYC binding protein 2	13q22	0.73
38642_at	MEMD	ALCAM	Y10183	Activated leukocyte cell adhesion molecule	3q13.1	0.73
35720_at	KIAA0893	WDR47	AB020700	KIAA0893 protein; WD repeat domain 47	1p13.3	0.72
38908_s_at	POLZ	REV3L	AL096744	REV3-like, catalytic subunit of DNA polymerase zeta (yeast)	6q21	0.72
35799_at	MDG1	DNAJB9	AL080081	DnaJ (Hsp40) homolog, subfamily B, member 9	14q24.2-q24.3	0.72
40631_at	TOB	TOB1	D38305	Transducer of ERBB2, 1	17q21	0.72
32212_at	AIF	PDCD8	AL049703	Programmed cell death 8	Xq25-q26	0.72
32807_at	BKLHD1; DKFZ	KBTBD2	AF004292	Kelch repeat and BTB (POZ) domain containing [illegible]	7p14.3	0.72
840_at	MOZ	ZNF220	U47742	MYST histone acetyltransferase (monocytic leukemia) 3	8p11	0.72
31823_at	CDP	CUTL1	M74099	Cut-like 1, CCAAT displacement protein	7q22.1	0.72
38741_at	Sec7p-L	PSCD2	U70728	Pleckstrin homology, Sec7 and coiled-coil domains 2	19q13.3	0.71
41821_at	CHED	CDC2L5	AA203246	Cell division cycle 2-like 5 (cholinesterase-related cell division controller)	7p13	0.71
980_at	NPC	NPC1	AF002020	Niemann-Pick disease, type C1	18q11-q12	0.71
38013_at	MTSG1	MTUS1	AL096842	Mitochondrial tumor suppressor 1	8p22	0.71
31847_at	D13S106E	C13orf22	X59131	Chromosome 13 open reading frame 22	13q12-q14	0.71
34903_at	MGC33190	ATXN7L4	AI017382	Ataxin 7-like 1	7q22.3	0.71
37910_at	RENBP; RBP; F	HCFC1	U52112	Chromosome X clone QLL-D9139, Qc-7G12, Qc-7C1, Qc-12B2, Qc-12D5, QLL-A074, Qc-9D3 map q28, complete sequence.	Xq28	0.71
41177_at	PFAAP3	FLJ12443	AW024285	Hypothetical protein FLJ12443	5p15.33	0.71
38270_at	PARG99	PARG	AF005043	Poly (ADP-ribose) glycohydrolase	10q11.23	0.71
40826_at	KP78	MARK3	M80359	MAP/microtubule affinity-regulating kinase [illegible]	14q32.3	0.71
453_at	BAF170	SMARCC2	U66616	SWI/SNF related, matrix associated, actin dependent regulator of chromatin, subfamily c, member 2	12q13-q14	0.71
38306_at	BIG1	ARFGEF1	AA477576	ADP-ribosylation factor guanine nucleotide-exchange factor 1(brefeldin A-inhibited)	8q13	0.70
36580_at	FLJ21795	FLJ13910	AL050139	Hypothetical protein FLJ13910	2p11.2	0.70
37047_at	NPC	NPC1	AF002020	Niemann-Pick disease, type C1	18q11-q12	0.70
35203_at	MORF	MYST4	AB002381	MYST histone acetyltransferase (monocytic leukemia) 4	10q22.2	0.70
36048_at	HRIHFB2436	ZFP318	AB015342	Zinc finger protein 318	6pter-p12.	0.70
33288_i_at	FPM315	ZNF263	D88827	Zinc finger protein 263	16p13.3	0.70
33404_at	CAP2	CAP2	U02390	CAP, adenylate cyclase-associated protein, 2 (yeast)	6p22.3	0.70
38343_at	KIAA0328	ALMS1	AB002326	Alstrom syndrome 1	2p13	0.70
40464_g_at	TRN	TNPO1	U70322	Transportin 1	5q13.2	0.70
39931_at	REDK	DYRK3	Y12735	Dual-specificity tyrosine-(Y)-phosphorylation regulated kinase 3	1q32.1	0.70
37788_at	MDC3	ADAM23	AF052115	A disintegrin and metalloproteinase domain 23	2q33	0.70
41355_at	BCL11A	BCL11A	N95229	B-cell CLL/lymphoma 11A (zinc finger protein	2p16.1	0.69
32655_s_at	SMAP	BRD8	X87613	Bromodomain containing 8	5q31	0.69
38994_at	CIS2; SSI2; Cis	SOCS2	AF037989	Suppressor of cytokine signaling [illegible]	12q	0.69

Affy ID	Synonyms	Symbol	Genbank	Description	Chromosome	fold difference
35648_at	KIAA0442	AUTS2	AB007902	Autism susceptibility candidate 2	7q11.22	0.69
33386_at	KBL; MGC2305	GCAT	Z97630	2-amino-3-ketobutyrate-CoA ligase	22q13.1	0.69
31801_at			AI808712	cDNA clone IMAGE:2359688		0.69
37915_at	DKFZp434H071	BAZ2B	AL080173	Bromodomain adjacent to zinc finger domain, 2B	2q23-q24	0.69
36025_at	KIAA0337	P164RHOGEF	AB002335	Rho guanine nucleotide exchange factor (GEF) 17	11q13.4	0.69
36612_at	KIAA0280	KIAA0280	D87470	KIAA0280 protein	11q13.4	0.69
33893_r_at	KIAA0470	KIAA0470	AB007939	KARP-1-binding protein	1q44	0.69
37537_at	ARFD1	ARFD1	L04510	Tripartite motif-containing 23	5q12.3	0.68
40215_at	UGCG	UGCG	D50840	UDP-glucose ceramide glucosyltransferase	9q31	0.68
36059_at	MEGF7	LRP4	AB011540	Low density lipoprotein receptor-related protein 4	11p11.2-p12	0.68
41460_at	RBM14	RBM14	AF080561	RNA binding motif protein 14	11q13.2	0.68
33427_s_at	ATRN	ATRN	AF106861	Attractin	20p13	0.68
32802_at	MARCH6	TEB4	AB011169	Similar to S. cerevisiae SSM4	5p15.2	0.68
39754_at	ITGB5	ITGB5	X53002	Integrin, beta 5	3q21.2	0.68
39845_at	OMI	PRSS25	AF020760	Protease, serine, 25	2p12	0.68
36588_at	KIAA0810	UNC84A	AB018353	Unc-84 homolog A	7p22.3	0.68
2054_g_at	NCAD	CDH2	M34064	Cadherin 2, type 1, N-cadherin (neuronal	18q11.2	0.68
131_at	TAF11	TAF11	X83928	TATA box binding protein (TBP)-associated factor, 28kDa	6p21.31	0.68
41000_at	FOXN3	CHES1	U68723	Checkpoint suppressor 1	14q24.3-q32.11	0.68
40780_at	CTBP2	CTBP2	AF016507	C-terminal binding protein 2	10q26.13	0.67
41049_at	HIRS-1	IRS1	S62539	Insulin receptor substrate 1	2q36	0.67
33932_at	GST1	GSPT1	X17644	G1 to S phase transition 1	16p13.1	0.67
844_at	PPP1R1A	PPP1R1A	U48707	Protein phosphatase 1, regulatory (inhibitor) subunit 1A	12q13.2	0.67
36005_at	SWAP2	SFRS16	AF042800	Splicing factor, arginine/serine-rich 16 (suppressor-of-white-apricot homolog, Drosophila)	19q13.3	0.67
37221_at	PRKAR2A	PRKAR2B	M31158	Protein kinase, cAMP-dependent, regulatory, type II, beta	7q22	0.67
32066_g_at	ICER	CREM	S68134	CAMP responsive element modulato	10p11.21	0.67
39219_at	IG/EBP-1	CEBPG	U20240	CCAAT/enhancer binding protein (C/EBP), gamma	19q13.11	0.67
39135_at	KIAA0767	DIP	AB018310	KIAA0767 protein; Death-inducing-protein	22q13.31	0.67
41431_at	KIAA0936	ICK	AB023153	Intestinal cell (MAK-like) kinase	6p12.3-p11.2	0.67
39992_at	BWR1-A	SLC22A1L	AF030302	Solute carrier family 22 (organic cation transporter), member 18	11p15.5	0.67
32065_at	ICER	CREM	S68134	CAMP responsive element modulato	10p11.21	0.67
40411_at	KIAA0181	NCOA6	D80003	Nuclear receptor coactivator 6	20q11	0.67
40710_at	CLGN	CLGN	D86322	Calmegin	4q28.3-q31.1	0.67
32444_at	RPL26	RPL26	X69392	Ribosomal protein L26	17p13	0.66
40151_s_at	PXR1	PEX5	Z48054	Peroxisomal biogenesis factor 5	12p13.3	0.66
40169_at	TIP47	M6PRBP1	AF057140	Mannose-6-phosphate receptor binding protein	19p13.3	0.66
32109_at	PLM	FXYD1	AA524547	FXYD domain containing ion transport regulator 1 (phospholemman)	19q13.1	0.66
34840_at			AI700633	CDNA: FLJ22642 fis, clone HSI06970		0.66
37650_at	ZNF127-Xp; RN	MKRN1	U41315	Makorin, ring zinc-finger protein; Escapes X chromosome inactivation; Ring zinc-finger protein (ZNF127-Xp)	7q34	0.66
39838_at	KIAA0622	CLASP1	AB014522	Cytoplasmic linker associated protein 1	2q14.2-q14.3	0.66
35467_g_at	DJ10	DNAJB12	W73046	DnaJ (Hsp40) homolog, subfamily B, member 12	10q22.1	0.66
33800_at	ADCY9	ADCY9	AF036927	Adenylate cyclase 9	16p13.3	0.66
41282_s_at	RNF69	PEX10	AA194159	Peroxisome biogenesis factor 10	1p36.32	0.65
38754_at	COM1	P8	AI557295	P8 protein (candidate of metastasis 1	16p11.2	0.65
41433_at	INCAM-100	VCAM1	M73255	Vascular cell adhesion molecule-1	1p32-p31	0.65
38568_at	TP53BPL	TOPORS	U82939	Topoisomerase I binding, arginine/serine-ric	9p21	0.65
32654_g_at	BRD8	SMAP	AW020536	Bromodomain containing 8	5q31	0.65
36506_at	AKAP450	AKAP9	AJ131693	A kinase (PRKA) anchor protein (yotiao) 9	7q21-q22	0.65
37951_at	DLC-1	DLC1	AF035119	Deleted in liver cancer 1	8p22	0.65
41789_r_at	KIAA0669	TSC22D2	AB014569	KIAA0669 gene product; TSC22 domain family, member 2	3q25.1	0.65
39792_at	hnRNP-R	HNRPR	AF000364	Heterogeneous nuclear ribonucleoprotein R	1p36.12	0.65
37451_at	SLC21A11	SLCO3A1	AL109695	Solute carrier organic anion transporter family, member 3A1	15q26	0.65
32696_at	PBX3	PBX3	X59841	Pre-B-cell leukemia transcription factor 3	9q33-q34	0.65
35344_at	KIAA0858	LMO7	AB020665	LIM domain 7	13q22.2	0.65
39646_at	MYSB	CACNB2	S60415	Calcium channel, voltage-dependent, beta 2 subunit	10p12	0.64
39000_at	NMT	NMT1	AF043324	N-myristoyltransferase 1	17q21.31	0.64
35367_at	CBP35	LGALS3	AB006780	Lectin, galactoside-binding, soluble, 3 (galectin 3	14q21-q22	0.64
34801_at	USP53	KIAA0710	AB014610	Ubiquitin specific protease 53	12q13.2-q13.3	0.64
40621_at	PAR-4	PAWR	U63809	PRKC, apoptosis, WT1, regulator	12q21	0.64
39723_at	CUL1	CUL1	AF062536	Cullin 1	7q36.1	0.64
39835_at	MTMR5	SBF1	U93181	SET binding factor 1	22q13.33	0.64
38712_at	CH1	C1orf9	AL035291	Chromosome 1 open reading frame 9	1q24	0.64
38794_at	NOR-90	UBTF	X53390	Upstream binding transcription factor, RNA polymerase I	17q21.3	0.64
36659_at	FLJ22259	COL4A2	X05610	Collagen, type IV, alpha 2	13q34	0.64
39295_s_at	ArgBP2a	ARGBP2	AF049884	Arg/Abl-interacting protein ArgBP2	4q35.1	0.64
35824_at	RP58	ZNF238	AJ223321	RP58 gene	1q44-qter	0.64
35741_at	Pip4k2B	PIP5K2B	U85245	Phosphatidylinositol-4-phosphate 5-kinase, type II, beta	17q12	0.64
38364_at	BCE-1	TLE4	AF068197	Transducin-like enhancer of split 4 (E(sp1) homolog, Drosophila)	9q21.31	0.64
41791_at	FLJ21040	MGC16824	AC002550	Esophageal cancer associated protein	16p12.3	0.64
38525_at	DKFZp564C047	STAM2	AF042273	Signal transducing adaptor molecule (SH3 domain and ITAM motif) 2	2q23.3	0.64
1269_at	GRB1	PIK3R1	M61906	Phosphoinositide-3-kinase, regulatory subunit, polypeptide 1 (p85 alpha)	5q13.1	0.64
41129_at	KIAA0033	TMEM41B	D26067	KIAA0033 protein; Transmembrane protein 41B	11p15.4	0.63
32248_at	MST107	PRO2730	AL045811	Hypothetical protein PRO2730	3p21.2	0.63
41466_s_at	pHZ-52	ZNF148	L04282	Zinc finger protein 148	3q21	0.63
38778_at	KIAA1046	HSHIN1	AB028969	HIV-1 induced protein HIN-1; KIAA1046 protein	4q31.21	0.63
38505_at	AGO2	EIF2C2	AL050151	Eukaryotic translation initiation factor 2C, 2	8q24	0.63
34822_at	ASPP2; 53BP2	TP53BP2	U58334	Tumor protein p53 binding protein, 2	1q42.1	0.63
37999_at	CPO	CPO	D16611	Coproporphyrinogen oxidase; Carboxypeptidase O	2q33.3	0.62
38812_at	LAMB2	LAMC1	X79683	LAMB2 mRNA for beta2 laminin.	1q31	0.62
34337_s_at	M96	MTF1	AJ010014	Metal response element binding transcription factor 2	1p22.1	0.62
36896_s_at	MOP3	ARNTL	AF044288	Aryl hydrocarbon receptor nuclear translocator-lik	11p15	0.62
38765_at	Dicer	DICER1	AB028449	Dicer1, Dcr-1 homolog (Drosophila	14q32.13	0.62
39857_at	STX11	STX11	AF044309	Syntaxin 11	6q24.2	0.62
38975_at	GENX-3414	GENX3414	AF062534	Genethonin 1	4q24-q25	0.61
35022_at	L-SOX5	SOX5	S83308	SRY (sex determining region Y)-box 5	12p12.1	0.61
41199_s_at	PSF	SFPQ	W27050	Splicing factor proline/glutamine rich (polypyrimidine tract binding protein associated)	1p34.3	0.61

Affy ID	Synonyms	Symbol	Genbank	Description	Chromosome	fold difference
36538_at	KIAA0771	PPP1R13B	AB018314	Protein phosphatase 1, regulatory (inhibitor) subunit 13B	14q32.33	0.61
32070_at	LPAP; CD45-AF	PTPRCAP	X97267	Protein tyrosine phosphatase, receptor type, C-associated protein	11q13.3	0.61
32587_at	TIS11D	ZFP36L2	U07802	Zinc finger protein 36, C3H type-like 2	2p22.3-p21	0.61
35683_at	ZCW3	KIAA0852	AB020659	Zinc finger, CW-type with coiled-coil domain 1	22q12.2	0.61
37312_at	TRIP-Br2	SERTAD2	D50917	SERTA domain containing 2	2p14	0.61
38001_at	hHT	CACNA1C	L04569	L-type voltage-dependent calcium channel a1 subunit	12p13.3	0.61
37156_at	LOC221810	ETV1	AF070641	Hypothetical protein LOC221810	7p21.3	0.60
40834_at	KIAA0300	PDZD2	AB002298	PDZ domain containing 3	5p13.3	0.60
38353_at	SPBC98	TUBGCP3	AF042378	Tubulin, gamma complex associated protein 3	13q34	0.60
36956_at	GLVR2	SLC20A2	L20852	Solute carrier family 20 (phosphate transporter), member 2	8p12-q21	0.60
39286_at	TOBL	TOB2	D64109	Transducer of ERBB2, 2	22q13.2-q13.31	0.60
39266_at			AF070632	Clone 24405 mRNA sequence		0.60
37698_at	PRKA1	AKAP1	X97335	A kinase (PRKA) anchor protein 1	17q21-q23	0.59
35821_at	HD3	HDAC3	U75697	Histone deacetylase 3	5q31	0.59
38086_at	EWI-3	IGSF3	AB007935	Immunoglobulin superfamily, member 3	1p13	0.59
38228_g_at	WS2A	MITF	AB006909	Microphthalmia-associated transcription facto	3p14.2-p14.1	0.59
32405_at	KIAA0707	THEA	AB014607	Thioesterase, adipose associated	1p32.3	0.59
39893_at	FLJ00058	GNG7	AB010414	Guanine nucleotide binding protein (G protein), gamma 7	19p13.3	0.59
36633_at	PKAP10	AKAP10	AA114830	A kinase (PRKA) anchor protein 10	17p11.1	0.59
39423_f_at	TEF2	SPOP	AJ000644	Speckle-type POZ protein	17q21.33	0.58
38005_at	SQV7L	SLC35D2	AJ005866	Solute carrier family 35, member D2	9q22.32	0.58
36658_at	KIAA0018	DHCR24	D13643	24-dehydrocholesterol reductase	1p33-p31.1	0.58
34817_s_at	A2LP	ATXN2L	U70671	Ataxin 2-like	16p11	0.58
33732_at	mu-ARP2	AP4M1	Y08387	Adaptor-related protein complex 4, mu 1 subun	7q22.1	0.58
32623_at	GABA-B R1a	GABBR1	AJ225028	Gamma-aminobutyric acid (GABA) B receptor, 1	6p21.31	0.58
37661_at	PMCA1	ATP2B1	J04027	ATPase, Ca++ transporting, plasma membrane 1	12q21.3	0.58
32833_at	CLK	CLK1	M59287	CDC-like kinase 1	2q33	0.58
41747_s_at	ADCAD1	MEF2A	U49020	MADS box transcription enhancer factor 2, polypeptide A (myocyte enhancer factor 2A)	15q26	0.57
34280_at	GABRE	GABRE	Y09765	Gamma-aminobutyric acid (GABA) A receptor, epsilon	Xq28	0.57
38175_at	KIAA0667	TIP120B	AB014567	TBP-interacting protein	3p25.2	0.57
34396_at	KIAA0978	ASXL1	AB023195	Additional sex combs like 1 (Drosophila	20q11.1	0.57
1137_at	GLVR2	SLC20A2	L20852	Solute carrier family 20 (phosphate transporter), member 2	8p12-q21	0.56
41329_at	FLJ10706	PACE-1	AI458463	Ezrin-binding partner PACE-1	1q24.2	0.56
35705_at	RVR	NR1D2	D16815	Nuclear receptor subfamily 1, group D, member 2	3p24.2	0.56
31526_f_at	TRE2	USP6	X63547	Ubiquitin specific protease 6 (Tre-2 oncogene)	17q11	0.56
38894_g_at	P40PHOX	NCF4	AL008637	Neutrophil cytosolic factor 4	22q13.1	0.56
36507_at	HUB1	ZNF282	D30612	Zinc finger protein 282	7q35-q36	0.56
39307_s_at	LCB	CLTB	X81637	Clathrin light chain b	5q35	0.56
38793_at	Troponin T	TNNT2	X74819	Troponin T2, cardiac	1q32	0.56
31843_at	KIAA0832	ESRRG	AB020639	Estrogen-related receptor gamma	1q41	0.56
38072_at	DJ465N24.2.1	DJ465N24.2.1	AL031432	Cone RP3-465N24 on chromosome 1p35.1-36.13	1p35.1-p36.13	0.56
35208_at	KIAA0874	ANKRD12	AB020681	Ankyrin repeat domain 12	18p11.22	0.55
40008_at	SCYA11	CCL11	U46573	Chemokine (C-C motif) ligand 11	17q21.1-q21.2	0.55
34018_at	COL19A1L	COL19A1	D38163	Collagen, type XIX, alpha 1	6q12-q13	0.55
32638_s_at	ATX	SMG1	AI610467	PI-3-kinase-related kinase SMG-1	16p12.3	0.55
39760_at	QK3	QK1	AL031781	Quaking homolog, KH domain RNA binding (mouse).	6q26-27	0.55
32167_at	MPPB	PMPCB	AF054182	Peptidase (mitochondrial processing) beta	7q22-q32	0.55
748_s_at	MXD2	MXI1	D63940	MAX interactor 1	10q24-q25	0.55
35314_at	KIAA0159	CNAP1	D63880	Chromosome condensation-related SMC-associated protein 1	12p13.3	0.54
558_at	KRT1A	KRT1	M98776	Keratin 1	12q12-q13	0.54
37208_at	CO9	PSPHL	AJ001612	Phosphoserine phosphatase-like	7q11.2	0.54
33290_at	MGC71303	INPP5B	M74161	Inositol polyphosphate-5-phosphatase, 75kD	1p34	0.54
857_at	PP2Calpha	PPM1A	S87759	Protein phosphatase 1A (formerly 2C), magnesium-dependent, alpha isoform	14q23.1	0.54
39971_at	LYL1	LYL1	M22637	Lymphoblastic leukemia derived sequence 1	19p13.2	0.53
39041_at	PDC-E2	DLAT	Y00978	Dihydrolipoamide S-acetyltransferase (E2 component of pyruvate dehydrogenase complex)	11q23.1	0.53
32129_at	RNF115	ZNF364	AL079314	Zinc finger protein 364	1q21.1	0.52
38886_i_at	ARHI	DIRAS3	U96750	Ras homolog gene family, member	1p31	0.52
33240_at	KIAA1095	SEMACAP3	AB029018	PDZ domain containing RING finger 3	3p13	0.52
41683_i_at	E1B	BCKDHB	U50708	Branched chain keto acid dehydrogenase E1, beta polypeptide (maple syrup urine disease)	6q13-q15	0.51
33253_at	KIAA0129	TRIM14	D50919	Tripartite motif-containing 14	9q22.33	0.51
142_at	hTAFII130	TAF4	U75308	TAF4 RNA polymerase II, TATA box binding protein (TBP)-associated factor, 135kDa	20q13.33	0.51
40217_s_at	CDS	CDS1	U65887	CDP-diacylglycerol synthase (phosphatidate cytidylyltransferase) 1	4q21.23	0.50
41788_i_at	KIAA0669	TSC22D2	AB014569	TSC22 domain family, member 2	3q25.1	0.50
39681_at	ZNF145	ZBTB16	AF060568	Kruppel-like zinc finger protein; Promyelocytic leukemia zinc finger protein (PLZF) gene.	11q23.1	0.50
32961_at	IRLB	MYCPBP	X63417	C-myc promoter binding protein	15q22.31	0.50
41503_at	KIAA0854	KIAA0854	AB020661	Zinc fingers and homeoboxes 2	8q24.13	0.50
35209_at	KIAA0766	EPM2AIP1	AB018309	EPM2A (laforin) interacting protein 1	3p22.1	0.49
39827_at	RTP801	DDIT4	AA522530	DNA-damage-inducible transcript 4	10pter-q26.12	0.49
34355_at	MeCP2	MECP2	AJ132917	Methyl CpG binding protein 2 (Rett syndrome	Xq28	0.49
39223_at	FLJ46354	FLJ46354	AL096749	FLJ46354 protein	1p32.3	0.48
40046_r_at	C18orf1	C18orf1	AF009426	Chromosome 18 open reading frame 1	18p11.2	0.48
32556_at	U2AF65	U2AF65	X64044	U2 (RNU2) small nuclear RNA auxiliary factor 2	19q13.42	0.47
40218_at	CDS	CDS1	U60808	CDP-diacylglycerol synthase 1	4q21.23	0.47
38599_s_at	XAP-5	DXS9928E	AD001530	DNA segment on chromosome X	Xq28	0.46
1052_s_at	NF-IL6-beta	CEBPD	M83667	NF-IL6-beta protein	8p11.2-p11.1	0.45
35303_at	INSIG1	INSIG1	U96876	Insulin induced protein 1	7q36	0.44
41685_at	KIAA0752	KIAA0752	AB018295	NY-REN-7 antigen	5q35.3	0.42
32127_at	BICD	BICD1	U90030	Bicaudal D homolog 1	12p11.2-p11.1	0.41
34843_at	KIAA0222	ZNF516	AL044599	Zinc finger protein 516	18q23	0.41
36739_at	PDK4	PDK4	U54617	Pyruvate dehydrogenase kinase, isoenzyme 4	7q21.3-q22.1	0.37
36680_at	AMY2B	AMY2B	M24895	Amylase, alpha 2B; pancreatic	1p21	0.37
36575_at	BL34	RGS1	S59049	Regulator of G-protein signalling 1	1q31	0.33
36254_at	TAC1	TAC1	U37529	Tachykinin, precursor 1 (substance K, substance P	7q21-q22	0.26
41260_at	P72	DDX17	U59321	DEAD (Asp-Glu-Ala-Asp) box polypeptide 17	22q13.1	0.20
